# Context-Dependent Risk Aversion: A Model-Based Approach

**DOI:** 10.3389/fpsyg.2018.02053

**Published:** 2018-10-26

**Authors:** Darío Cuevas Rivera, Florian Ott, Dimitrije Markovic, Alexander Strobel, Stefan J. Kiebel

**Affiliations:** ^1^Chair of Neuroimaging, Faculty of Psychology, Technische Universität Dresden, Dresden, Germany; ^2^Chair of Differential and Personality Psychology, Faculty of Psychology, Technische Universität Dresden, Dresden, Germany

**Keywords:** risk aversion, decision making, active inference, modeling, single subject

## Abstract

Most research on risk aversion in behavioral science with human subjects has focused on a component of risk aversion that does not adapt itself to context. More recently, studies have explored risk aversion adaptation to changing circumstances in sequential decision-making tasks. It is an open question whether one can identify evidence, at the single subject level, for such risk aversion adaptation. We conducted a behavioral experiment on human subjects, using a sequential decision making task. We developed a model-based approach for estimating the adaptation of risk-taking behavior with single-trial resolution by modeling a subject's goals and internal representation of task contingencies. Using this model-based approach, we estimated the subject-specific adaptation of risk aversion depending on the current task context. We found striking inter-subject variations in the adaptation of risk-taking behavior. We show that these differences can be explained by differences in subjects' internal representations of task contingencies and goals. We discuss that the proposed approach can be adapted to a wide range of experimental paradigms and be used to analyze behavioral measures other than risk aversion.

## Introduction

It is typically assumed that humans, as well as other animals, prefer courses of action free of risk and uncertainty; e.g., when foraging for food, easier and safer patches are preferred (Kacelnik and Bateson, 1996; Myerson et al., 2003). However, this safety-seeking behavior is highly contextual: In many situations, the course of action with the least risk or least uncertainty is not the one that can best fulfill the current goals. For example, while an animal might choose to go for small, easy prey at the beginning of a day, toward the end, if sustenance is not ensured for the night with the small prey, bigger prey must be sought, with all the risks it entails (McNamara and Houston, 1992; Kacelnik and Bateson, 1996).

Personality traits related to such risk proneness and risk aversion are well-studied in humans. However, it is known that, as with animals, the preference toward risky or safe choices is not a static parameter of behavior; instead, this preference seems to change to better fit the context. While much research has been conducted on these dynamic context effects in animal behavior (Caraco et al., 1980, 1990; Cartar and Dill, 1990; Kacelnik and Bateson, 1996) and in anthropology and related fields (Winterhalder and Smith, 2000), relatively few studies seem to have explored the same themes in human subjects (Kolling et al., 2012; Mobbs et al., 2013; Houston et al., 2014).

A possible reason for this is that studying the dynamics of risk aversion in an individual is notoriously difficult (Kellen et al., 2016). This is mostly due to the fact that risk aversion must be measured, by definition, in a situation in which risk and uncertainty play a great part. In these situations, the behavior of the subject is stochastic (Rieskamp, 2008), which may complicate the analysis of the behavioral data.

To work around this difficulty, experimenters have resorted to experimental manipulations to indirectly assess or directly ask for the subject's preferences or uncertainty (Hey and Orme, 1994), which comes with its own set of pitfalls (Charness et al., 2013). Other methods are based on averaging behavior across many decisions, sometimes across many subjects and tasks, and inferring how behavior changes, on average, as a function of context, e.g., (Kolling et al., 2014; Economides et al., 2015; Schwartenbeck et al., 2015; Walasek and Stewart, 2015; Kellen et al., 2016). This approach has the downside of being blind to subject- and/or trial-specific changes on choice preference.

Here we performed a model-based analysis of the adaptation of risk aversion to context based on subject-specific behavioral responses. To do this, we combined a behavioral model formulated in the recently-developed active inference framework (Friston et al., 2015) with maximum-likelihood estimators of subject-specific parameters. The behavioral model allowed us to estimate a subject's preference for risk at every decision. Importantly, the proposed method has the advantage of not requiring multiple observations of the same context in order to estimate a subject's preferences, instead harnessing statistical power from every decision made across all trials.

As an experimental proof of principle, we applied the resulting model-based technique to a sequential decision-making task first presented in Kolling et al. (2014). In this task, subjects must make a sequence of decisions to accumulate points toward a target. This task is well-suited to study the effects of context on risk aversion, as the risk of not reaching the target varies throughout the sequence of decisions, thereby prompting subjects to adapt their choices to the current risk context.

With this approach, we find inter-subject differences in the way that context modulates risk aversion, as well as motivational and confidence-related differences in the way subjects evaluate a context and make a decision.

## Methods

### The task

Subjects performed a game-like task first introduced in Kolling et al. (2014), in which they have a total of eight trials (decisions) to accumulate points, in what we call a mini-block. For each mini-block, a threshold is set, of which subjects are informed; if at the end of the mini-block the number of points accumulated does not exceed the threshold, all points for that mini-block are lost. The overarching goal for the subjects is to accumulate as many points as possible over all mini-blocks.

In every trial, the subjects are presented with two choices; they can either choose (i) an action that yields a small number of points with a high probability of success, or (ii) one which yields a high number of points, but with a low probability of success. Each yields zero points if it does not win. We call these two choices the “safe choice” and the “risky choice,” respectively. Following (Kolling et al., 2014), there are eight pairs of a safe and risky choices (called action pairs; see Table 1), and they are presented in random order in every mini-block, without repetition. The order is not known to the subjects. Subjects are informed that the outcomes of the two bets at each trial are randomly selected, independently of each other.

**Table 1:** The eight action pairs offered to subjects in each mini-block, in random order.

	**Risky option**				**Safe option**			
	**Prob. of success**	**Reward**	**Expected value**	**Subj. value**	**Prob. of success**	**Reward**	**Expected value**	**Subj. value**
1	0.35	265	92.75	0.243	0.9	100	90	−0.449
2	0.35	260	91	0.130	0.6	180	108	0.491
3	0.45	240	108	0.980	0.9	115	103.5	0.134
4	0.45	190	85.5	−0.149	0.6	150	90	−0.677
5	0.35	245	85.75	−0.208	0.75	145	108.75	0.215
6	0.2	350	70	0.210	0.55	145	79.75	−0.123
7	0.4	245	98	0.442	0.75	170	127.5	0.118
8	0.3	210	63	−0.165	0.9	120	108	0.329

*Each row represents a single action pair with one safe option and one risky option. The column “Prob. of success” indicates the probability of getting the reward and the column “Reward” indicates the magnitude of a reward in points. The column “Expected value” lists the probability of success times the reward. The column “Subj. value” shows the subjective value (see main text for the definition)*.

The maximum possible number of points in a mini-block is 2,005, which is achieved only in the unlikely case when the subject chooses and wins every risky option. The threshold to be reached, for each mini-block, is pseudo-randomly chosen from four possibilities: 595, 930, 1,035, and 1,105.

**Figure 1:**
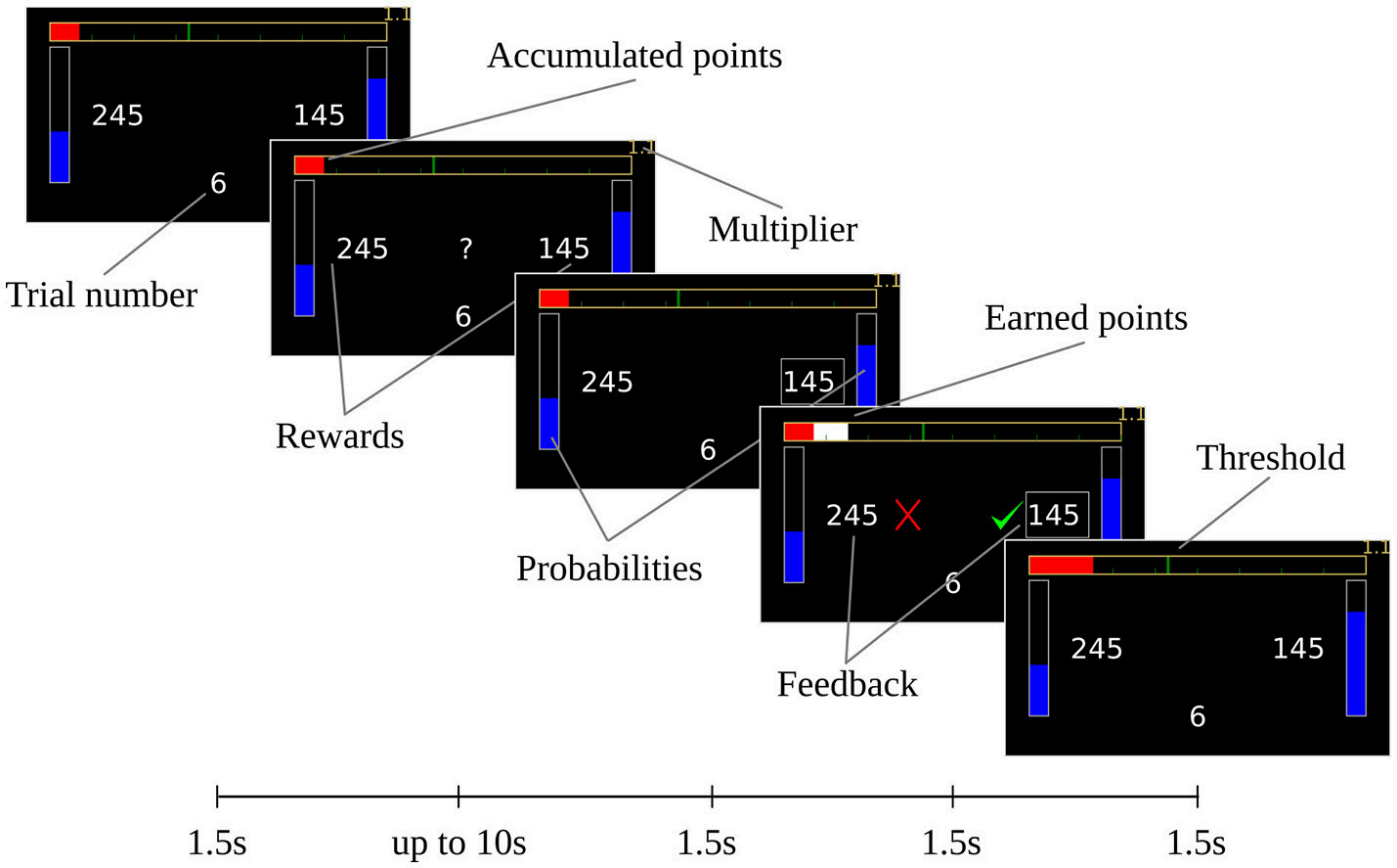
Experimental design. The information was shown to subjects on a computer screen. Each screen was shown for 1.5 s, except for the decision screen (the second screen), which was displayed either for a maximum of 10 s or until the subject made a decision. If no decision was made during the 10 allotted seconds, another screen (not shown) reminds the subject to stay attentive, and the trial is repeated. After the final screen is shown for 1.5 s, a new trial begins. At the end of each mini-block (of eight trials), the subject is informed about success or failure of reaching the threshold, i.e., the required number of points for this mini-block. For visual clarity, the elements are shown bigger in this figure than in the experiments.

In this task, the variables relevant to making a decision are the trial number, the number of points accumulated so far in the mini-block, the current threshold and the presented action pair. All this information is shown to the subject on the screen during a trial (see Figure 1). On the top of the screen, a bar shows the subjects how many points they have accumulated through the current mini-block, as well as the threshold. We used a bar to prevent subjects from calculating exactly how many points were needed, in order to maintain uncertainty in the decisions. The two available actions are shown on the left and right of the screen; the position of the two choices (right or left side) was randomized. The probabilities of success for the actions are displayed as vertical bars, and the reward magnitudes are displayed as numbers. The trial number of the ongoing mini-block is displayed on the bottom.

Subjects made choices by pressing the *X* or *M* keys on a standard keyboard, to choose the option on the left or right, respectively. After a decision was made, feedback was displayed that informed the subjects whether the two choices had been successful or not (regardless of which choice was made). If the selected action was successful, the points were added to the top bar in white. At the beginning of the next trial, the white bar turns the same color as the rest of the bar. At the end of the mini-block, subjects were informed as to the number of points gained in that mini-block. The timings of the screens can be seen in Figure 1.

Following (Kolling et al., 2014), a mini block-specific multiplier was used, which can be seen in the top-right corner of the display. This number is set according to the current threshold, with values 1.1, 2.3, 3.3, and 4.2 (for the corresponding thresholds in ascending order). If the subject goes past the threshold of this mini-block, the accumulated points are multiplied by this multiplier. This is done to offset the difficulty of the higher thresholds and maintain the subject's motivation.

For this task, we defined the context as the combination of trial number, action pair (offered), number of points that have been earned so far, and threshold for the current mini-block. These are the variables that are relevant to making a decision and may prompt subjects to adapt their behavior in a specific context. For example, subjects may decide differently about a specific action pair when late in a mini-block with many points required to surpass the threshold, as compared to early in the mini-block. We use this definition of context throughout this paper.

The task was performed by 35 subjects, recruited from a pool of subjects at the Technische Universität of Dresden. 13 were men and 22 women, with an average age of 26 years (standard deviation 4.6). All had normal or corrected-to-normal eyesight.

The training session consisted of 4 mini-blocks, each with a different threshold. After the training session, each subject completed a total of 48 mini-blocks, 12 with each threshold, in randomized order. The session was divided into two blocks with 24 mini-blocks each, with a pause between the two blocks, totaling around 45 min per subject, depending on the time they took for each decision. Both the offers and their outcomes were chosen randomly before the experiments and were used with all subjects. The position of the risky option (left or right hand side of the screen) was randomly chosen for each trial.

The payout was of 10€, not tied to performance. While some studies have found evidence that real vs. hypothetical payouts affect behavior in gambling tasks (Locey et al., 2011; Xu et al., 2018), it is unclear if and how adding non-hypothetical rewards would affect behavior in our task, as risky and safe choices can both be valid ways of winning a mini-block. Relatedly, whether monetary incentives alleviate or completely eliminate these biases is still under investigation, with studies attempting to eliminate the effects of these biases seeing mixed effects (Fantino et al., 2007; Locey et al., 2011). Moreover, incentivizing subjects has been found to exacerbate certain cognitive biases and hurt performance (Camerer and Hogarth, 1999; Hertwig and Ortmann, 2001).

The study was approved by the Institutional Review Board of the Technische Universität Dresden (protocol number EK 541122015) and conducted in accordance with the declaration of Helsinki. All subjects gave their written, informed consent.

### Active inference model

Our behavioral model is based on active inference, as was described in Friston et al. (2015). In this and the following sections, we briefly describe the Active Inference framework, as well as the generative model parameters that we used, and the fitting procedures to fit the model to each subject's data.

The active inference agent chooses actions which minimize expected free energy (i.e., maximize model evidence or minimize surprise), given the generative model and the goals of the task. The generative model is a formal description of the agent's knowledge about the existing hidden states of the environment and the existing rules that define transitions between these states. Model inversion based on Bayes' theorem allows us to formulate the agent's beliefs about the current and past states of the world, and to generate expectations about the future.

In practice, exact probabilistic inference is rarely computationally tractable, as generative models capture complex task dynamics. Hence, one often has to resort to an approximate inference scheme when defining model inversion. Active inference is based on the so-called variational approximation which allows treating posterior beliefs over specific hidden variables as conditionally independent from other factors of the hidden states space (Beal, 2003; Daunizeau et al., 2009). This method allows us to obtain closed-form algebraic equations that define the evolution of beliefs over the hidden states of the world.

In active inference, the problem of choosing an action that best fits the goals of the task is cast as an inference problem. In fact, actions are taken as another hidden state (so-called control states) of the environment, and as such benefit from the same simplification that the closed-form update equations bring.

In the following sections, we discuss the underlying hidden Markov model in which the task contingencies are represented. Full details of the mathematical derivation of the update equations can be found in Friston et al. (2015).

### Generative model

The full generative model of active inference is built with the following (Friston et al., 2015):

- A set of observations
- A set of hidden states and actions
- A generative model over observations, states and actions
- An approximate posterior probability over hidden states

In what follows, we describe these required components of the generative model in more detail.

#### Observations and hidden states

We take the hidden states of the environment to be two-dimensional. The first dimension is that of the accumulated points: it describes how many points in the mini-block the agent has won so far. These are taken to be the integers in the interval (0, 200), where 0 means that no points have been so far earned, and 200 is the maximum number of points possible. In the experiments, the points run from 0 to 2005; however, for the sake of computational efficiency, we divided the points by 10 and rounded up. In trial simulations, we found that this approximation made no difference in the behavior of the agent, and it allowed us to simulate the task in computation times adequate for our fitting procedures. Additionally, we did not include all 200 states (points) in the model, but rather created a cut-off point shortly after threshold; this allowed us to further reduce the computation times without affecting the results. The value chosen was 1.2 times the threshold for most of the computation, except for the (much smaller) computations in the section Risk preferences for the low- and high-STP groups, where it was set at 1.5 times the threshold to fully accommodate the consequences of all action pairs. Very few observations were made by subjects beyond the cut-off point (a maximum of 12 out of 384 for one subject) and they were removed from the pool.

The second dimension of the hidden states is the current offer; the action pairs are labeled from 1 to 8, as in Table 1.

We have chosen the observation likelihood, which defines dependence of observations on hidden states, as an identity matrix. This was to reflect the fact that, during the experiments, subjects receive direct observations pertaining to the number of points so far accumulated and the current offer. This means that the observations are fully informative about the state of the environment; hence the inference about the current state corresponds to matching beliefs to observations.

#### Generative model of states and action

The state transition probability defines the agent's belief about the evolution of the environment, both as a consequence of the agent's actions and of the passing of time.

We now describe the agent's generative model pertaining to the task at hand that consists of accumulating points throughout the trials. It is beyond the scope of this work to explain how an agent (or subject) comes to build (learn) this generative model. Here we assume that subjects have learned an accurate representation of the environment, which would correspond to setting the agents' generative model to correspond to the true generative process of the environment (i.e., the exact transition rules of the environment). This reflects the fact that the rules of the task are simple and well-explained to the subjects at the beginning of the experiment.

The evolution of the environment is described in terms of transition matrices. There is one for each of the available actions. In our model, there are two actions available: risky and safe. The effect of each action depends on the current offer, which is why it is included as a dimension of the hidden states. The matrices can be represented with the following equation:

(1) $$B_{\text{s/r}}\left(X_{t}+R_{\text{s/r},\;j},\;X_{t}\right)=P_{\text{s/r},\text{succ},j}$$

(2) $$B_{\text{s/r }}\left(X_{t},\;X_{t}\right)=P_{\text{s/r},\text{ fail},\text{ j}}$$

(3) $$B_{\text{s/r}}\left(X,\;X_{t}\right)=0,\;\forall X\notin \left\{X_{t}+R_{s/r,\text{ j}},\;X_{t}\right\}$$

Where *B*_*s*/*r*_ is the transition matrix for the safe or risky action, respectively, *X*_*t*_ is the current number of points, and *P*_*r*/*s, succ*/*fail, j*_ and *R*_*r*/*s, j*_ are the probabilities of success/failure and the reward of the j-th action pair, respectively. These reflect the rules of the game, in which choosing an action can either yield that action's reward, with the action's probability, or yield no reward.

Regarding the current offer (action pair), we chose a generative model which differs from the generating process of the environment. In the environment, transitions from one offer to another are randomized, but no action pair is repeated during a single mini-block. However, because keeping track of the offers already seen (and those still to come) is a very costly process, the agent instead believes that all transitions (i.e., from the current action pair to all others) are equally probable. We believe that this generative model is more likely to resemble that of human subjects.

#### Prior preferences over goal states

The goals of the task are stated as a distribution over the last state, after the eighth trial. The agent will make its decisions by comparing its predictions regarding states to be visited in the future by making a set of decisions, and the prior distribution over the last state.

In our case, this distribution describes the relative desirability of outcomes of a mini-block, in terms of the accumulated points. A score is assigned to each of these hidden states of the environment; the higher this score, the more the agent will seek to be in this state by the end of the mini-block. We assumed that subjects learned this distribution through both task instructions and experience during training.

**Figure 2:**
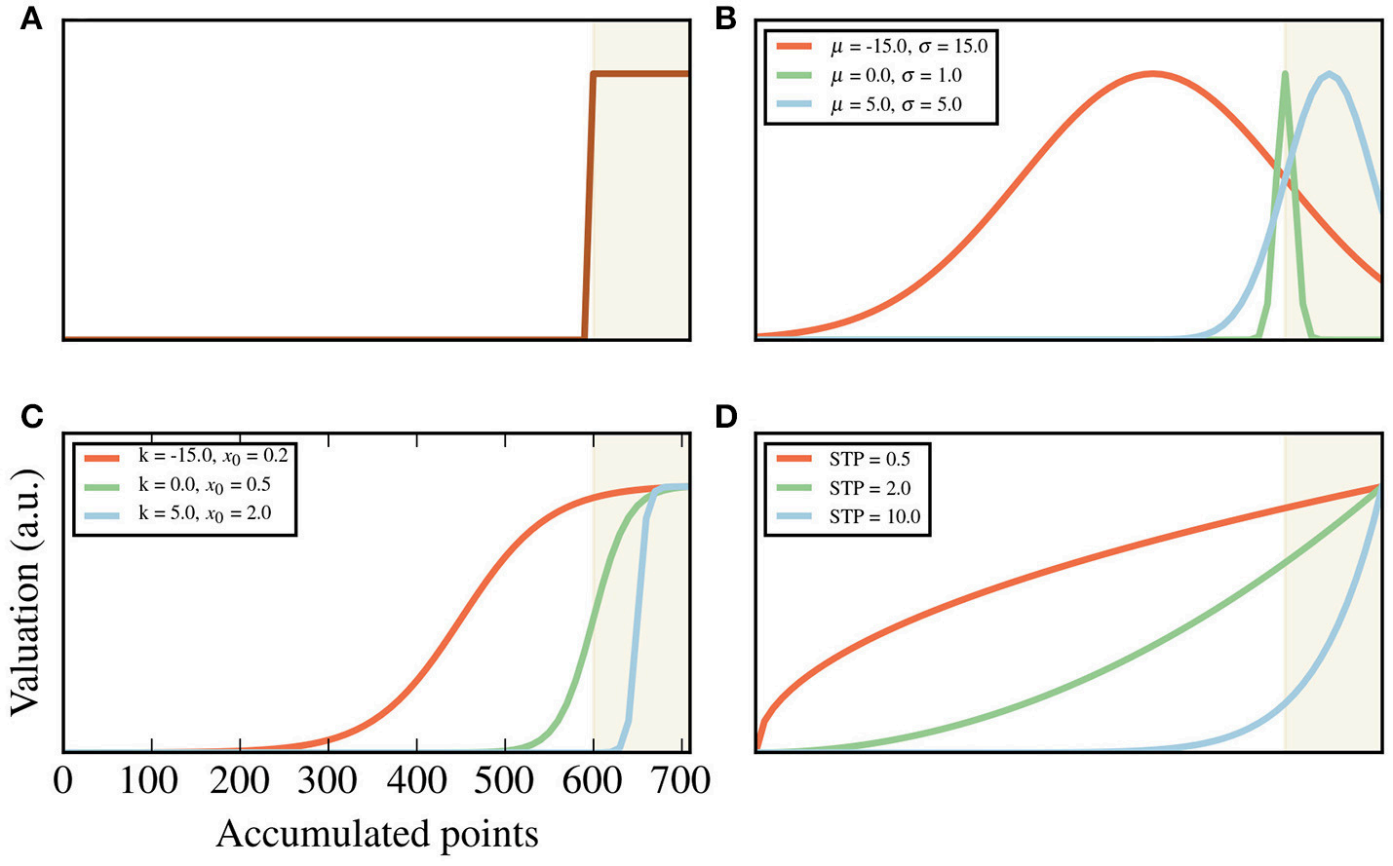
Goal shapes. Goals are expressed as a valuation of each of the relevant hidden states, which in this case are the accumulated points after the 8th trial. These valuations are presented here as shapes, following the equations described in the Methods section. The shade areas are those points above threshold. For visualization, only points from 0 to 700 are plotted, with a threshold of $\hat{x}=595$. **(A)** An example of a shape that follows the task instructions to the letter. All points below threshold have a value of 0, while all above are valued the same. **(B)** Gaussian, **(C)** sigmoid and **(D)** exponential families of shapes. For each family, three examples are plotted (shown in different colors), with their corresponding parameters shown in the legend. The values of μ and κ are relative to the threshold $\hat{x}$. For example, μ = −150 represents a mean located at $\hat{x}-\mu =445$. In **(D)**, STP stands for sensitivity to points, which is the parameter of the exponential family (see the section “Subject-specific parameter values” above).

Given the task instruction one could reason that the best functional form for the preferences is the one with zeros everywhere below threshold and some positive number above threshold (see Figure 2A). However, it is important to note that the prior preferences over the goal state do not necessarily reflect the rules of the game, but rather establishes the behavioral strategy that the agent believes is the best for winning. Therefore, we expect that, through experience, subjects build different beliefs as to what the best way to win is. Because of this, we allowed for subject-specific goal distributions.

For our fitting procedures, we used three distinct shapes for the goals: Gaussian, sigmoid, exponential. By changing their parameters, we were able to generate a large family of shapes that are consistent with the task instructions. In particular, the sigmoid family recreates the task instructions to the letter, such that any state below threshold has no value, and a ramping up holds for those above threshold. Examples of these shapes can be seen in Figures 2B,C.

The sigmoid family has two parameters: slope and center. The center determines where the transition from zero to one is centered, and the slope parameter is the slope at the center. These follow the equation:

(4) $$f_{\text{sig}}(x)=\;\frac{1}{1+\;e^{-m(x-x_{0})}}$$

Where *m* and *x*_0_ are the slope and the center, respectively. A very high *m* and a *x*_0_ at threshold reproduces the step function as in Figure 2A.

The exponential family has only one parameter, the coefficient of the exponent. This family follows the equation:

(5) $$f_{\exp}\left(x\right)=\;e^{\kappa x}$$

where κ is a free parameter, For the remainder of this work, we will refer to the parameter κ of the exponential goal shape as sensitivity to points (STP), in analogy to the sensitivity to delay/probability in the discounting literature (Basile and Toplak, 2015). This family describes a “ramping-up,” which does not incorporate the existence of a threshold. This shape represents a simple heuristic, where the agent assigns exponentially larger amounts of preference to higher number of points.

Finally, the Gaussian family has two parameters: mean and standard deviation. They are given by the equation:

(6) $$f_{g}\left(x\right)=Ae^{-\frac{{\left(x-\mu \right)}^{2}}{2\sigma^{2}}}$$

where μ and σ are the mean and standard deviation, respectively, and *A* is the normalization constant. This family reflects a compromise between the general rule of “more points are better” and a strong preference to states close to threshold (depending on the mean of the distribution).

Note that, while most of the shapes considered do not have a strong threshold that the agent must surpass (with the exception of the sigmoid, when the slope is high), they do not conflict with the rules of the task; by maximizing points (which can be said of any monotonously-increasing shape), a mini-block can be won. Additionally, certain parameter ranges (for example, higher values for the exponential) do include the idea of soft thresholds, i.e., that the agent has a strong preference for ending above threshold.

#### Posterior over actions (update equations)

To calculate the posterior probability distribution over the available actions at each trial, *P*(*a*_*t*_|*s*_*t*_, *m*), our model makes use of the following equation:

$$\log P\left(a_{t}|s_{t}\right)=F\left(s_{t},\;C\right)+\log\left(\beta_{a_{t}}\right)$$

where β_*a*_*t*__ is a subject-specific choice bias, which does not depend on the context and the function *F(s*_*t*_, _*C*_*)* compares the projected future states to the agent's goals *C*; see Friston et al. (2015) for details.

**Figure 3:**
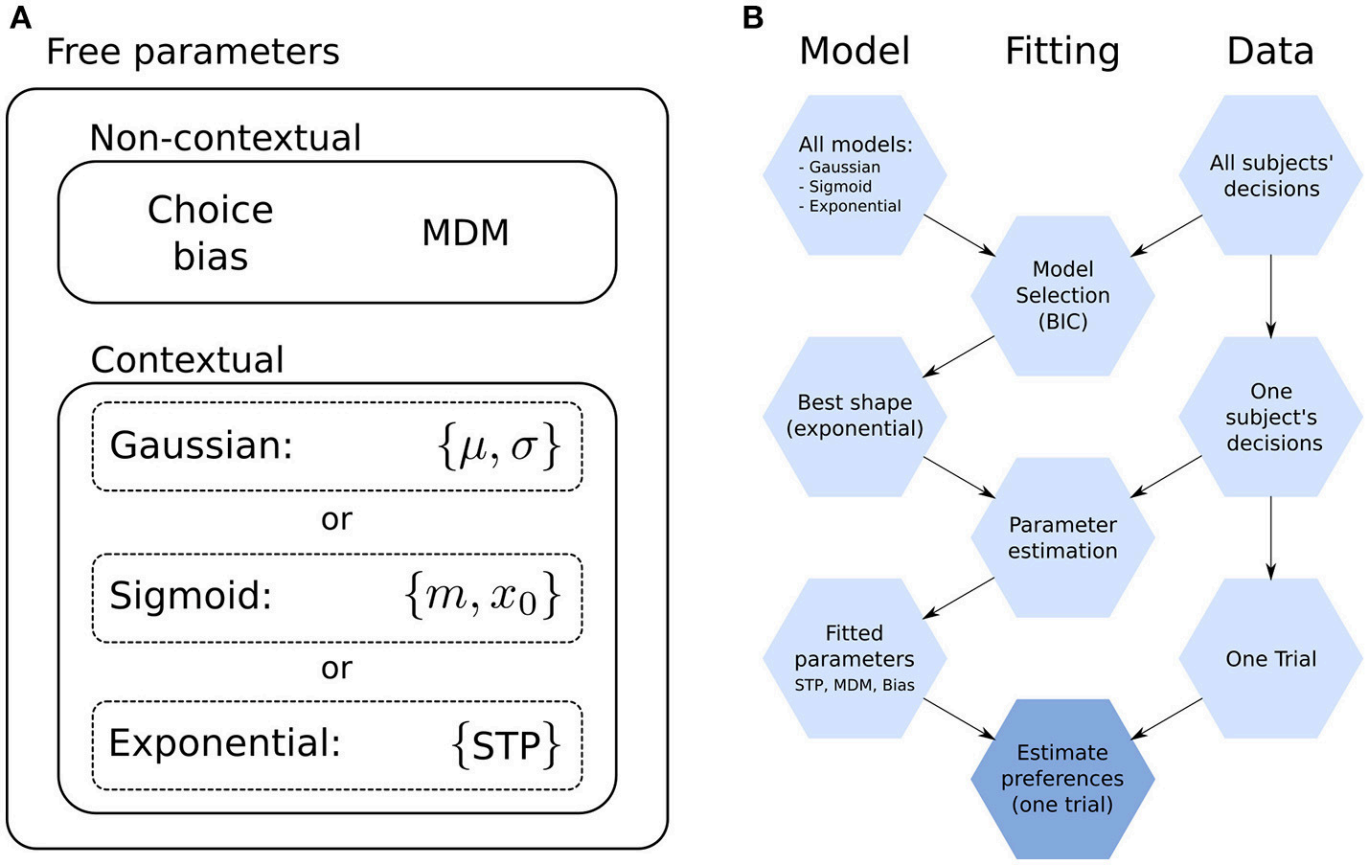
Model parameters and fitting. **(A)** Model parameters. The goal shape (e.g., exponential) is shared by all subjects, while the rest (Subject-specific, free parameters) are fitted to each subject. Non-contextual parameters are those that affect all decisions equally (the choice bias and the maximum decision multiplier, MDM). Contextual parameters are those whose effect on decisions changes with context, which are those related to the shape of the goal. The number of parameters depends on the shape; the Gaussian has two (μ, σ), the sigmoid two (*m, x*_0_) and the exponential one (STP) (see “Prior preferences over goal states”). In total, the model with the exponential has four free parameters and with Gaussian and sigmoid five. **(B)** Diagram for the proposed method to estimate subjects' preferences. The process starts at the top, with the collection of all models and the experimental data for all subjects. Nodes on the left-hand column are models, which become more refined as the process moves downwards. The same is true for the Fitting and Data columns (center and right-hand, respectively). The final step, “Estimate preferences,” is repeated for each trial, while the rest are performed only once.

We introduced this choice bias parameter because we observed a marked average preference of most subjects for the safe choice across the entire data set (see “Standard analysis of behavioral data” below). Choice bias is a prior preference for or against the risky option, regardless of context and, more specifically, of the current offer. That is, β_*a*_*t*__ has two components, β_*risky*_ and β_*safe*_. The values referred to in the main text are those of β_*risky*_. This parameter complements the goal parameters by adding a component that is non-contextual on top of the contextual goal parameters. For an overview of the model parameters, see Figure 3A.

### Fitting the model

Our model has free parameters which we fitted to every subject independently by doing a grid-search over the relevant part of the parameter space. These parameters are of two categories, affecting the agent in different ways, see Figure 3A.

In the first category, the first parameter affects how extreme the probability distributions from which actions are sampled can be. We call this parameter maximum decision modifier (MDM) and it corresponds to the α parameter in Friston et al. (2015). The effect of the MDM is that, for a low value, the distributions approach 50/50, regardless of context, while for large value, they approach either 0/100 or 100/0, depending on the context. This can be interpreted as controlling how certain the agent can be about its decisions; its value, either optimal (performance-wise) or inferred (fitted to a subject) is both task- and subject-dependent. The second parameter in this category is choice bias, which is a non-contextual number added to (or subtracted from) the preference for risk.

The second category pertains to the shape of the goal distribution. These are the three families discussed above, each with its own parameters.

For each subject, we performed a grid-search over the parameter space and calculated, for each set of values for the parameters, the data likelihood of the model. Through this procedure, we created a likelihood map for each subject, which represents a multi-dimensional probability distribution over parameter values.

The parameters we searched are as follows. For the Gaussian family, μ from $\hat{x}-15$ to $\hat{x}+15$, σ from 1 to 15, where $\hat{x}$ is the threshold, both with increments of 1. For the sigmoid family, we chose centers *x*_0_ between $\hat{x}-15$ to $\hat{x}+15$ with increments of 1, and slope *m* from 0.1 to 3 with increments of 0.1. We chose to set the values of μ and *x*_0_ to be centered about threshold in order to unify the parameter values across all conditions and simplify calculations; since the interpretation of the fitted agents is made in terms of their shapes and not their parameter values, this choice has no effect on the results. For the exponential family, the parameter STP was taken from 0.5 to 10, in increments of 0.1. Smaller increments in these parameters caused no discernible differences in the posteriors over actions.

The parameter MDM was searched in the range 0.1 to 5 in increments of 0.1, and from there to 60 in increments of 5. The range of MDM is segmented in this way because for higher values (>5), small increments create no discernible differences in the posterior distributions over actions.

Finally, the choice bias parameter was searched between 0.1 and 2 in increments of 0.1. Maximum likelihoods rapidly drop after 1.2, and by 2 are already many orders of magnitude lower.

### Model comparison

We made use of the Bayesian information criterion (BIC) (Schwarz, 1978) to evaluate each model and compare between them, using the guidelines in Kass and Raftery (1995).

In Figure 3B we show a diagram of the entire procedure comprising model comparison, parameter fitting and preference estimation (which will be discussed below).

## Results

The influence of the environment, as well as the internal state of an agent, e.g., a foraging bee, on the decision-making process under varying risk has been extensively studied in the fields of behavioral ecology (for animals) and behavioral anthropology (for humans; Winterhalder and Smith, 2000). In these studies, animal or human subjects are placed in environments, or presented with tasks, in which a sequence of decisions must be made in order to reach an overarching goal. For each decision, two or more options are presented to the subject, differing in how risky these options are, i.e., how likely it is that they will yield no reward, and how much reward, e.g., food or money, they may yield. Here we define risk as the probability that a chosen action does not yield any reward, when these probabilities are known to the decision-making agent (as opposed to uncertainty, where the contingencies are unknown). Because this definition of risk (and its usage in risky/safe choices) refers to the differences on the probabilities of success for the two choices in a trial, “relative risk” would be a more adequate name; however, since it is the only definition of risk used in this study, we have dropped the adjective “relative” from the name for simplicity.

For this work, to exemplarily showcase this approach, we made use of one such task, introduced in Kolling et al. (2014), in which the variations in the context, i.e., the states of the environment and of the agent relevant to the decision-making process, allowed us to study the effects of context on the choices of human subjects. More specifically, it allowed us to study the adaptation of risk aversion to changing context, as has been done with animals (Caraco et al., 1980, 1990; Cartar and Dill, 1990; Kacelnik and Bateson, 1996).

This section is divided into three parts: firstly, we show, using standard analysis tools, the behavioral results. Secondly, we present a computational model for the task, as well as the results from fitting this model to behavioral data. Finally, we show that our approach can be used to track the change in subjects' risk aversion throughout the experiment.

### Standard analysis of behavioral data

We first performed the same data analysis as was performed in Kolling et al. (2014) on our data set of 35 subjects.

**Figure 4:**
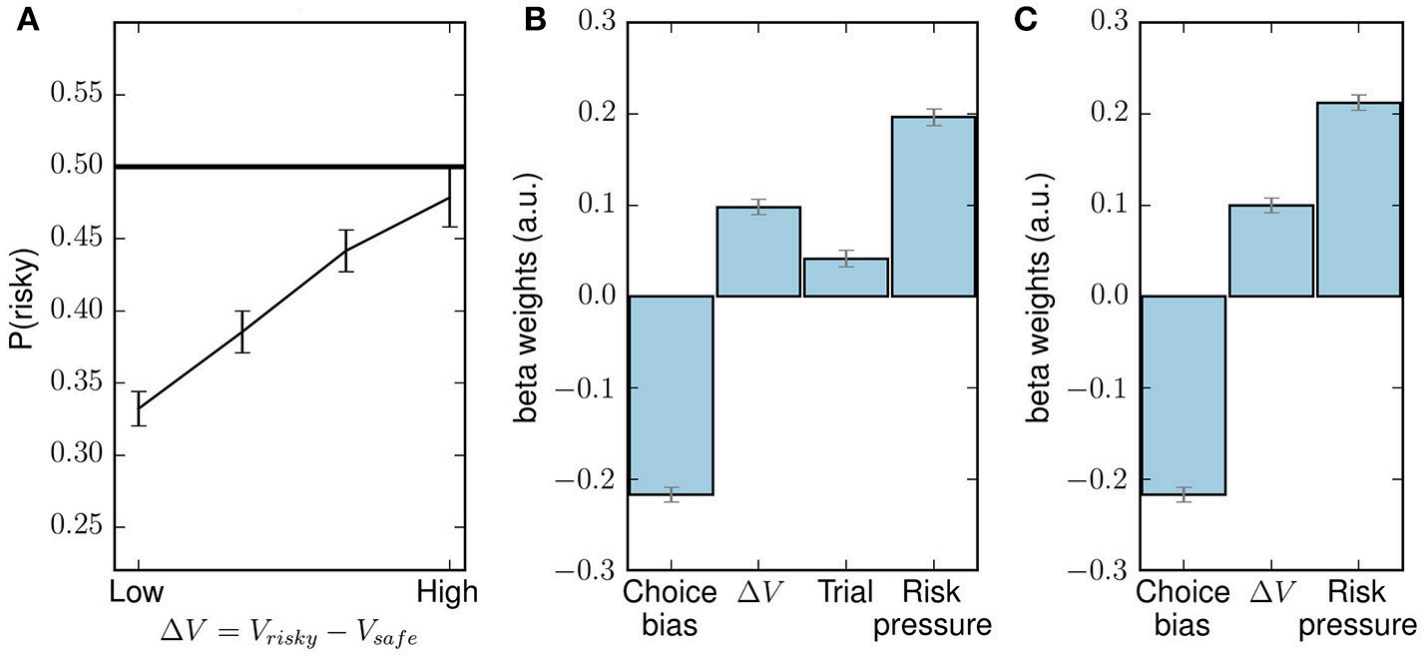
Behavioral results which reproduce the results reported in Kolling et al. (2014). **(A)** Probability of choosing the risky choice, P(risky), as a function of Δ*V*, the difference between the values of the risky choice and the safe choice, averaged across all subjects. **(B)** Fixed-effects logistic regression with decisions pooled from all subjects as the dependent variable (1 for risky, 0 for safe) and four regressors: a constant term (choice bias), the difference in values for the two choices [as in (A)], trial number and risk pressure. **(C)** GLM regression as in (B), but without the trial number as regressor. Error bars are SEM. All regressors in (B,C) are significant (*p* < 0.001).

In Figure 4A, we show the average probability of choosing the risky action as a function of Δ*V*, the difference in subjective values of the risky and safe choices. To reproduce the findings in Kolling et al. (2014), we defined the subjective value of a choice as:

(7) $$V_{\text{risky/safe }}=\;{\tilde{p}}_{\text{risky/safe}}(\text{win }|\text{offer})+{\tilde{r}}_{\text{risky/safe}}(\text{offer})$$

where $\tilde{p}$ and $\tilde{r}$ are the probability of success and the reward for the current offer, where offers are normalized by the mean and standard deviation across all offers. The tildes in $\tilde{p}$ and $\tilde{r}$ indicate that they have been normalized: for each offer, we subtracted the mean across all offers and divided by the standard deviation (see Table 1). Note that the term “subjective” refers to the fact that it differs from the objective definition of expected value (probability multiplied by reward); Kolling et al. (2014) opted for this definition as they found that it better fitted subject's choices. Note that, to show the expected value in the conventional sense, Table 1 lists the standard expected values for each offer pair, based on a multiplication of probability and reward size.

As already found in Kolling et al. (2014), it can be seen that, when averaged across all subjects and all mini-blocks, the probability of choosing the risky offer increases monotonously with the difference in subjective values. Note that Δ*V* does not offer a full description of a context, for it does not take into account the potential pressure created by the upcoming end of the mini-block or the necessity to go above threshold in the task (Kolling et al., 2014).

Kolling et al. (2014) defined the variable “risk pressure” as a more detailed (but still incomplete; see below) description of the context of the present task. Risk pressure is defined as the average number of points the subject would need to earn in each of the remaining trials in the mini-block in order to surpass the threshold:

(8) $$\Gamma_{t}=\{\begin{array}{cc} \frac{\hat{x}-x_{t}}{T-t} & x_{t}<\hat{x}\\ 0 & \text{otherwise} \end{array}$$

where *t* is the current trial number, $\hat{x}$ is the threshold, *x*_*t*_ the accumulated number of points at *t*, and *T* the total number of trials. They found evidence, using a regression analysis, that its value is a predictor of subjects' choices. Risk pressure has values in the interval [0, $\hat{x}$]: (i) zero when points are above-threshold, (ii) and $\hat{x}$ when no points have been earned and only one trial is left. In the present task, risk pressure values above 350 represent a context in which the subject cannot possibly win, as the highest possible offer is 350 (see Table 1).

We replicated the behavioral results in Kolling et al. (2014) using two fixed-effects generalized linear models (GLMs); both assume the same regressor values for all subjects. The first model had four regressors, namely: a constant term (choice bias), Δ*V*, trial number and risk pressure. The results can be seen in Figure 4B. In our case, probably due to the larger number of subjects, all regressors are significant (*p* < 0.001); however, to exactly reproduce the previous results we created a second GLM with three regressors: constant term, Δ*V* and risk pressure. The resulting regression can be seen in Figure 4C.

### Adaptability of risk aversion

In this section, we use a simple method to analyze the adaptation of risk pressure, where many trials are binned together and averaged to calculate the subject's preferences for risk aversion for this set of trials. This is an obvious extension of the analysis performed in Kolling et al. (2014) for Δ*V* (also see Figure 4A). We show the limitations of such a method and show, in subsequent sections, how the model-based approach we present in this paper sidesteps these difficulties.

When trying to estimate subjects' preferences from a single decision in a stochastic environment, that single decision is not necessarily representative of the subject's preference. This is especially true when the preference is not too strong, which we found to be the case for this task. In such a case, to estimate the small size of the preference, one would require many exposures to this decision in the same context. However, this is suboptimal due to potentially confounding memory effects (Mather et al., 2003; Bornstein et al., 2017): the subject may remember having made a specific context-dependent decision before, which will influence any subsequent repetitions.

In order to study the context dependence of risk aversion, a simple analysis method is to make use of risk pressure as a description for the context: We binned risk pressure to obtain enough decisions to calculate a risk pressure-dependent risk aversion value for each bin.

Any such reduction of dimensionality will have the disadvantage of possibly mapping two very distinct contexts onto the same risk pressure value. For example, a risk pressure of 300 on the last trial, when an option to win 350 points has been offered to the subject, presents a context with 20% probability of going above threshold (see Table 1) if the subject takes the risky offer, and 0% if the subject takes the safe offer. The same risk pressure near the beginning of a mini-block would present a game in which winning is almost impossible, and choice becomes inconsequential. This downside is inevitable in order to obtain reliable estimates on the mean risk aversion when using averaging methods.

**Figure 5:**
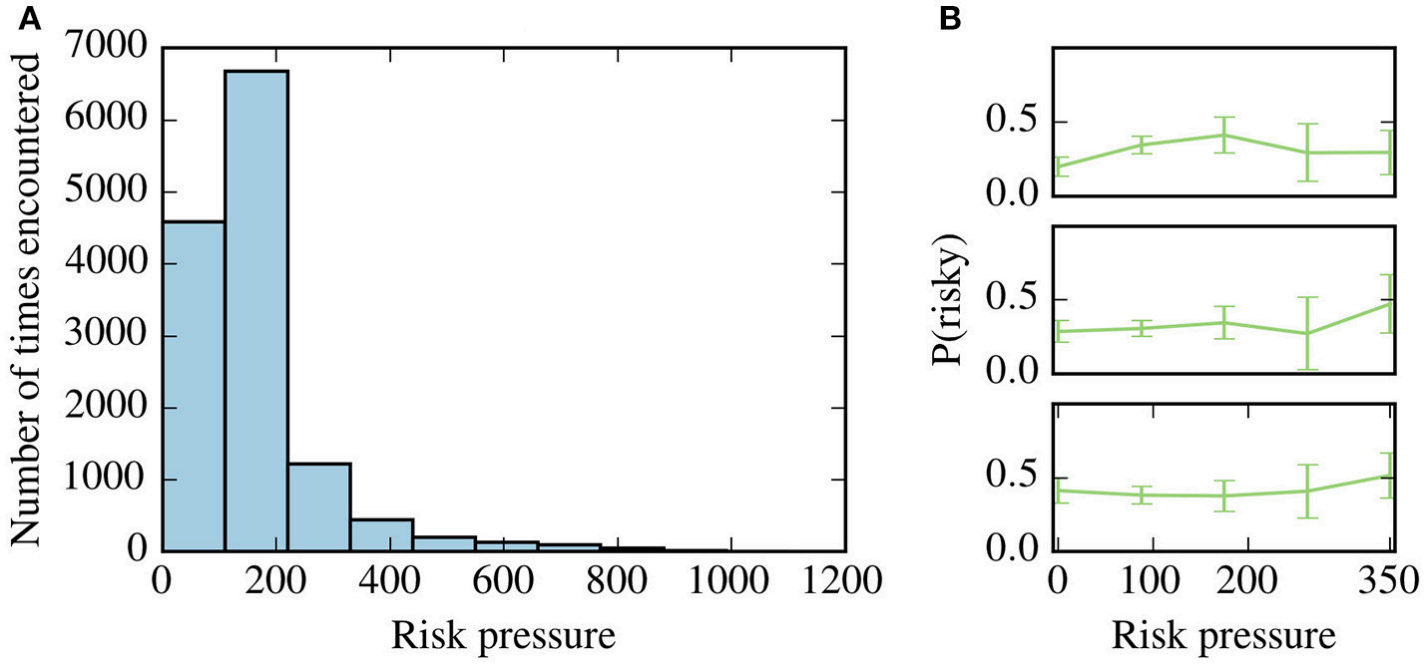
Risk pressure and risk aversion. **(A)** Histogram of the values of risk pressure encountered by all subjects. **(B)** Adaptive risk aversion for three representative subjects where we plot, against risk pressure, the frequency that the subject chooses the risky option, P(risky), calculated by averaging over all choices for each subject, binned according to the context in which the choice was made. Only risk-pressure values between 0 and 350 are shown as only few contexts were observed in higher values of risk-pressure by all subjects–see **(A)**.

As can be seen in Figure 5A, high values of risk-pressure above 350 were observed much more rarely than lower values. This is a direct consequence of the task design: if values of risk pressure above 350 were commonly observed, subjects might lose motivation due to the high difficulty of the task.

We calculated risk aversion as a function of risk pressure, by binning the contexts for each subject according to values of risk pressure. For each bin, the average risk aversion can be calculated by averaging across all decisions made in the bin. In Figure 5B, the frequency of choosing the risky choice for each bin is shown for three representative subjects, along with the 95% confidence intervals. The results for the remaining subjects can be seen in the supporting information (Supplementary Figure 1). We found that for 25 out of 35 subjects, the null hypothesis that risk aversion is constant for all values of risk pressure cannot be rejected (one-way ANOVA, *p* > 0.05).

In what follows, we introduce a novel method for inferring the risk aversion on a trial-by-trial basis.

### Model-based approach

In this section we describe a novel method for estimating the subject's preference (i.e., risk aversion) for every context they observed. This method takes advantage of all decisions made by the subject to calculate the subject's preference in any given context. We introduce a model-based approach using active inference, with free parameters that we fitted to each subject using all the decisions made by the subject (see Methods). This so-called agent (the fitted model) can then be used to estimate, for any given context, the probability distribution over actions that is most consistent with the subject's entire set of choices.

This process is equivalent to finding the underlying mechanism with which subjects make their decisions (which is assumed not to change throughout the experiment). Once this mechanism has been found, it can be used to calculate the preferences (risky vs. safe) that the subject had for any trial.

We will show that these fitted agents can be used to study in greater detail the changes of risk aversion in human subjects.

#### Model parameters and fitting

We fitted three different models which differed in their parameterization of the goal shape; see Methods. For each of these three models, we fit the model's parameters to the choices made by the subjects throughout all the mini-blocks. We fitted a total of four parameters to subjects' choices: (i) the maximum decision multiplier (MDM), (ii) up to two parameters that control the shape of the goal distribution, and (iii) a choice bias parameter. For more details on these parameters, see Methods.

The MDM and choice bias parameters differ from the goal shape parameters in that their effect on choices does not depend directly on the context. For this reason, their effects will be discussed mostly separately from those of the goal shape parameters.

#### Modeling inter-subject differences

We first hypothesized that previously observed inter-subject differences in risk aversion (Choi et al., 2007; Shead and Hodgins, 2009) can be modeled for this task here as differences in the internal representation of the task contingencies. For example, an agent for whom finishing below threshold is unacceptable (Figure 2A) will tend to display, if necessary, riskier behavior than one who is simply trying to increase the number of points (blue and brown line in Figure 2).

To address this hypothesis, we tested for inter-subject differences in the model parameters that best fit the behavior of each subject. To do this, we compared two models: With the first, we inferred a single, best-fitting parameter set for the data of all subjects, i.e., same parameter values for all subjects. For the second model, we inferred subject-specific parameters. We found that the BIC difference between these two models strongly favored the subject-specific model (ΔBIC > 100), as per the guidelines in Kass and Raftery (1995).

Given that there was strong evidence for inter-subject variability in how subjects represented the goal of the task, we next performed a model comparison between the three goal shapes, to determine whether one family was significantly better at explaining subjects' behavior than the other two.

We found strong evidence that the exponential shape is better at explaining subjects' behavior than the other two families, as evidenced by ΔBIC > 100, and therefore all the following results were obtained with this exponential shape family.

Finally, we tested whether there was also evidence for condition-specific differences in goals, i.e., different goal shapes for the four different thresholds, for each subject. However, we found no significant advantage in separating data into conditions; therefore, all results that follow were obtained with the subject-specific model but with the same parameters across conditions.

We also found that the introduction of the subject-specific MDM significantly improved model fit (ΔBIC > 100). Adding the choice bias parameter further increased model fit (ΔBIC > 100).

To summarize, we found very strong evidence for the model with inter-subject differences, with the exponential family being the best model for representing subjects' goal shapes. This model was used for the results presented in what follows.

### Subject-specific parameter values

**Figure 6:**
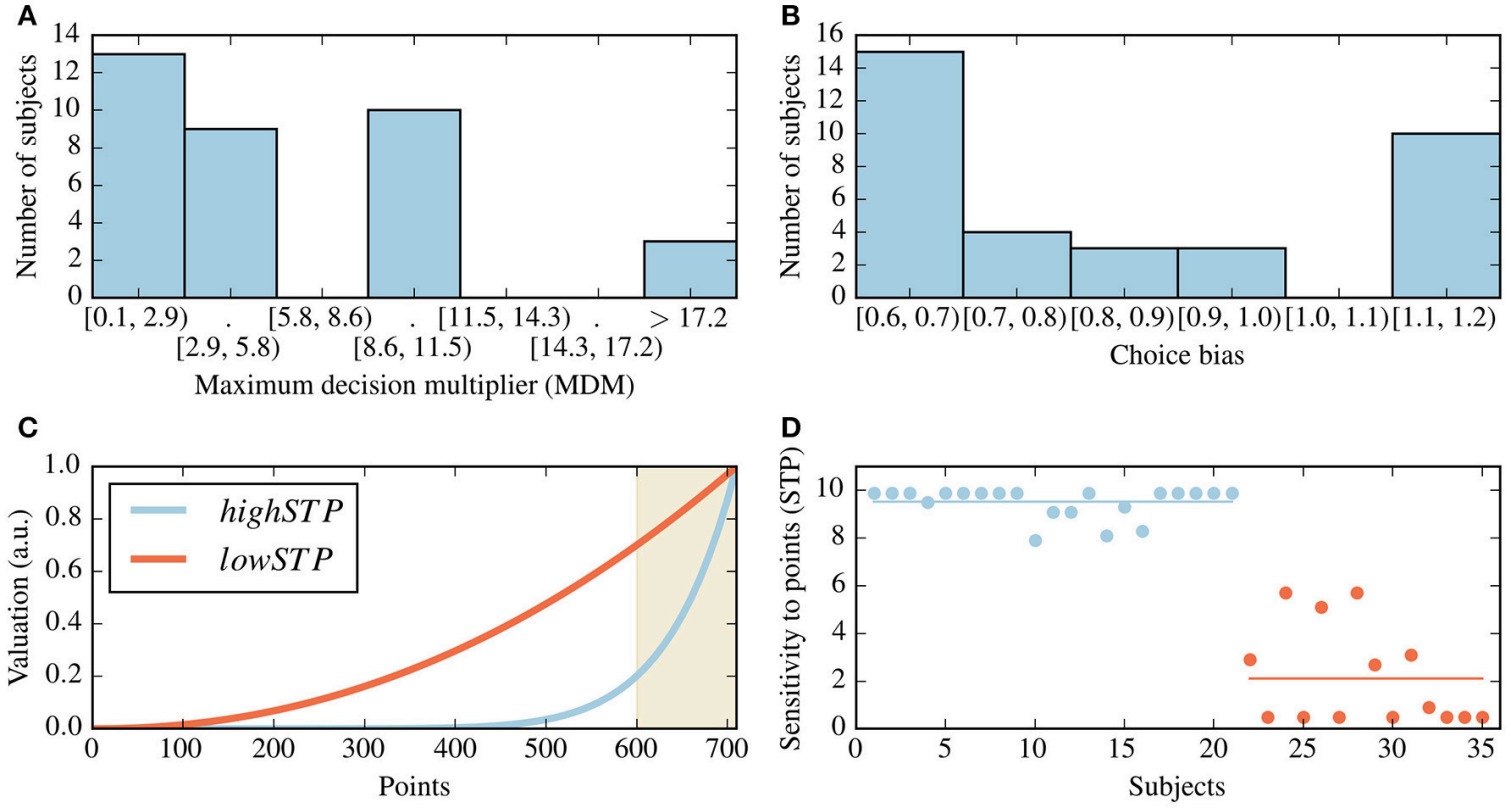
Summary of estimated parameters for all subjects. **(A)** Histogram for the best-fit values of the maximum decision multiplier (MDM) for all subjects. **(B)** Histogram of the best-fit choice bias parameter values, where the value of 1 stands for unbiased choice behavior and values smaller than one for a bias for the safe choice. **(C)** The shapes elicited by the centers of each of two identified clusters, as given by the cluster center's sensitivity to points (STP) value. The shaded area indicates above-threshold values (depicted here with a threshold of 595). **(D)** Scatter plot of the STP parameter for all subjects. The colors indicate cluster membership, as in **(C)**. Subjects were sorted by cluster membership. The horizontal lines represent the values of STP which were determined as the cluster centers in **(C)**.

The MDM values we obtained for different subjects range from 0.1, which makes most decisions close to a 50/50 decision, to values as high as 20, which creates preferences close to being deterministic. In Figure 6A we present a histogram of the values of MDM for all subjects, where the average MDM value is 6.01 (standard deviation of 5.70). The number of bins and their positions were determined using the SciPy (Jones et al., 2001) implementation of the Freedman-Diaconis estimator (Freedman and Diaconis, 1981), assuming equal-width bins.

The low values of the MDM for many subjects reflect a degree of indecisiveness, with which decisions are mostly driven by the choice bias parameter; this seems to be at least partially due to the fact that the task rarely presents contexts in which one action is clearly better than the other. Those subjects with a high MDM tend to have many strong preferences throughout a mini-block; these strongly determined decisions are the least affected by the choice bias.

All best-fit choice bias parameter values were found between 0.6, which favors safe choices, to 1.2, which favors risky choices (a value of 1 has no effect), and a mean of 0.8 (see Figure 6B). For most subjects (26 out of 35), we found a best-fit choice bias parameter smaller than one, which indicates an increased preference for the safe choice.

We also found inter-subject differences in the shape parameters, reflected in subject-specific values for the STP. To summarize these results, we clustered all subjects based on their fitted STP using the k-means algorithm (Lloyd, 1982; Goutte et al., 1999). We found that the best number of clusters is 2, and the clusters resulting from this procedure can be classified as low- or high-STP; see Figures 6C,D. Subjects belonging to the low-STP group implement a simple heuristic of accumulating more points, regardless of threshold; those in the high-STP group are best described by representing rather sharp goal shapes, i.e., by giving relatively low importance to points below threshold. For a full list of the inferred parameters, see the (Supplementary Table 1).

Finally, we calculated a Pearson cross-correlation matrix for the fitted parameters and found no significant correlations between them.

### Recovering subjects' preferences

With the estimated parameters for each subject, we can build a decision-making agent (henceforth, a fitted agent) which makes decisions the most consistently with its corresponding subject. Thus, for each subject we have a fitted agent.

Using these subject-specific fitted agents, we recovered subjects' preferences via the posterior distributions over actions that the subject-specific fitted agents calculate for every context. The key advantage of this procedure is that it eliminates the necessity of having many trials measured experimentally in the same context (or the same variable of interest, which is related to context, e.g., risk pressure), when investigating subjects' trial-by-trial preferences and their dynamics. In other words, the model-based approach is like a “mathematical microscope” (Moran et al., 2013) which enables the experimenter to replace actual (here binary) choice observations of a single subject, for a specific context, by inferred posterior probabilities lying continuously between 0 and 1.

### Adaptation of risk aversion

In this section we show the behavior of the fitted agent in a similar manner as that used for Figure 5B, discuss the shortcomings of this approach and, in the next section, show how our model-based approach can be used to sidestep these shortcomings.

We first looked at the subject-specific general risk aversion, i.e., how likely subjects are to pick the safe option throughout all contexts they observed. Averaged across all the trials and subjects, we observed a probability of choosing the risky option of 0.41, with a standard deviation of 0.07 across subjects, before taking into account the choice bias parameter. Despite the differences in parameter values, we found no significant differences between subjects (one-way ANOVA, *p* > 0.05).

**Figure 7:**
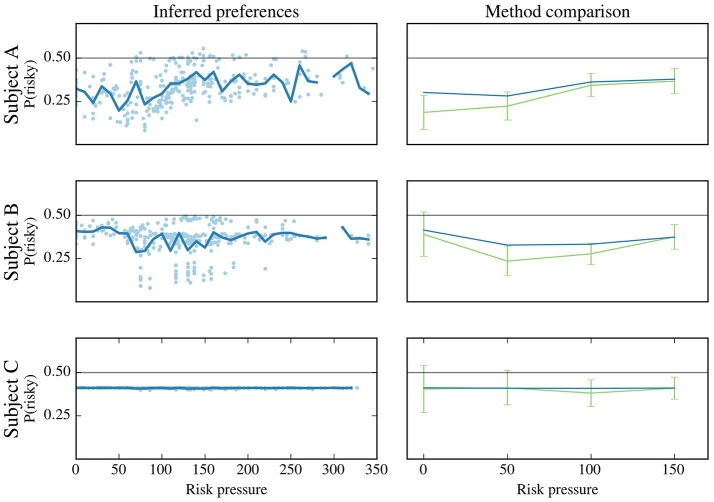
Probability of choosing risky option for three subjects. The subjects are labeled **(A–C)**, and each row corresponds to one subject. These subjects are the same as those used in Figure 5B. Left-hand column: plot for each subject, labeled “Inferred preferences,” represents the subject's preference for the risky option. For each data point (context of being offered, under as specific value of risk pressure, a specific option pair, see Table 1) the subject's preference for the risky option (y-axis) is plotted against risk pressure (x-axis). For each risk-pressure value, the average preference for the risky option is calculated (solid line). Not all risk pressures have been sampled by the subjects during the experiment as can be seen from the broken solid line for subjects **(A,B)**. Right-hand column: to validate the model-based approach, we show the binning method shown in Figure 5 (green) and the preferences estimated by the model-based approach, averaged on the same bins as the binning method. Note that the definition of bins deviate from Figure 5 as only risk pressure values up to 150 are shown in these plots; this is done for a better comparison with the model-based approach (see main text).

Figure 7 (left), where we show the recovered preferences for three representative subjects (the same as in Figure 5B), for the set of all the contexts that they observed (for the rest of the subjects, see Supplementary Figure 2). Note that the spread of the dots is not measurement noise but the inferred preferences for differences offers. While different trends can be seen for the three representative subjects (e.g., subject A's preference for risk increased for the first 100 units of risk pressure, while subject B's preference dipped in the 50–150 range), this representation suffers from similar problems as those discussed in section “Adaptability of risk aversion.”

Relatedly, the sharp vertical swings of the averages in Figure 7 (left-hand side, solid lines) are due only to the fact that the observations made by subjects do not sample all values of risk pressure evenly; the calculation of the preferences (vertical positions of the dots) itself entails no stochasticity, as the model is deterministic. These variations make extracting any significant information from these plots all but impossible. However, our model-based approach allows us to overcome this difficulty by being able to predict how such a subject would behave in the “missing” contexts, uncovering the differences in behavior between subjects A and B in greater detail; See the next section.

For validation purposes, we compared the estimates obtained with our method and with the binning method (see Figure 5B). Since most of the observed contexts (for all subjects) are for low values of risk pressure (see Figure 5A), and to allow for a better comparison between the methods, we binned the decisions in risk pressure values between 0 and 200 (with bins at 0, 50, 100, and 150). The results are shown in the right-hand column of Figure 7, alongside the average obtained with our method (the same as the left-hand column), using the same bins as the binning method, i.e., we binned the preferences estimated by our method and calculated their average for each bin. For clarity, in the remainder of this work, we use the name “model-based binning” to refer to the results presented in the right-hand column of Figure 7, i.e., to binning the model-based risk aversion to calculate averages (blue line). For all three subjects both methods agree in their estimate of how often, in a specific risk pressure range, these three subjects will choose the risky choice. There are small differences, especially for subjects A and B, which we will discuss below.

### Risk preferences for the low- and high-STP groups

In this section, we show that the jaggedness of the average risk preference for each value of risk pressure (Figure 7, left-hand column) is mostly caused by the biases introduced by the set of contexts observed by the subjects. We also show how one can use the fitted agents to “see through” these biases, to gain further insight into the adaptation of risk aversion to context for the two groups of subjects.

For the results in this section, we used the agents that were fitted to subjects A and B from Figure 7. To facilitate a direct comparison between the risk aversion adaptations of these two subjects, we set their MDM to 20 and their choice bias to 1. Because the effects of these two parameters are not context-dependent, the principled findings in this section are not affected by using the fitted values while the visual clarity of the figures is increased. Note that for both subjects, MDM values are similar in their effects (10 and 5, respectively) and choice biases are identical (0.6). We used these two modified agents to extrapolate these subjects' preferences to contexts that were not observed during the experiment.

**Figure 8:**
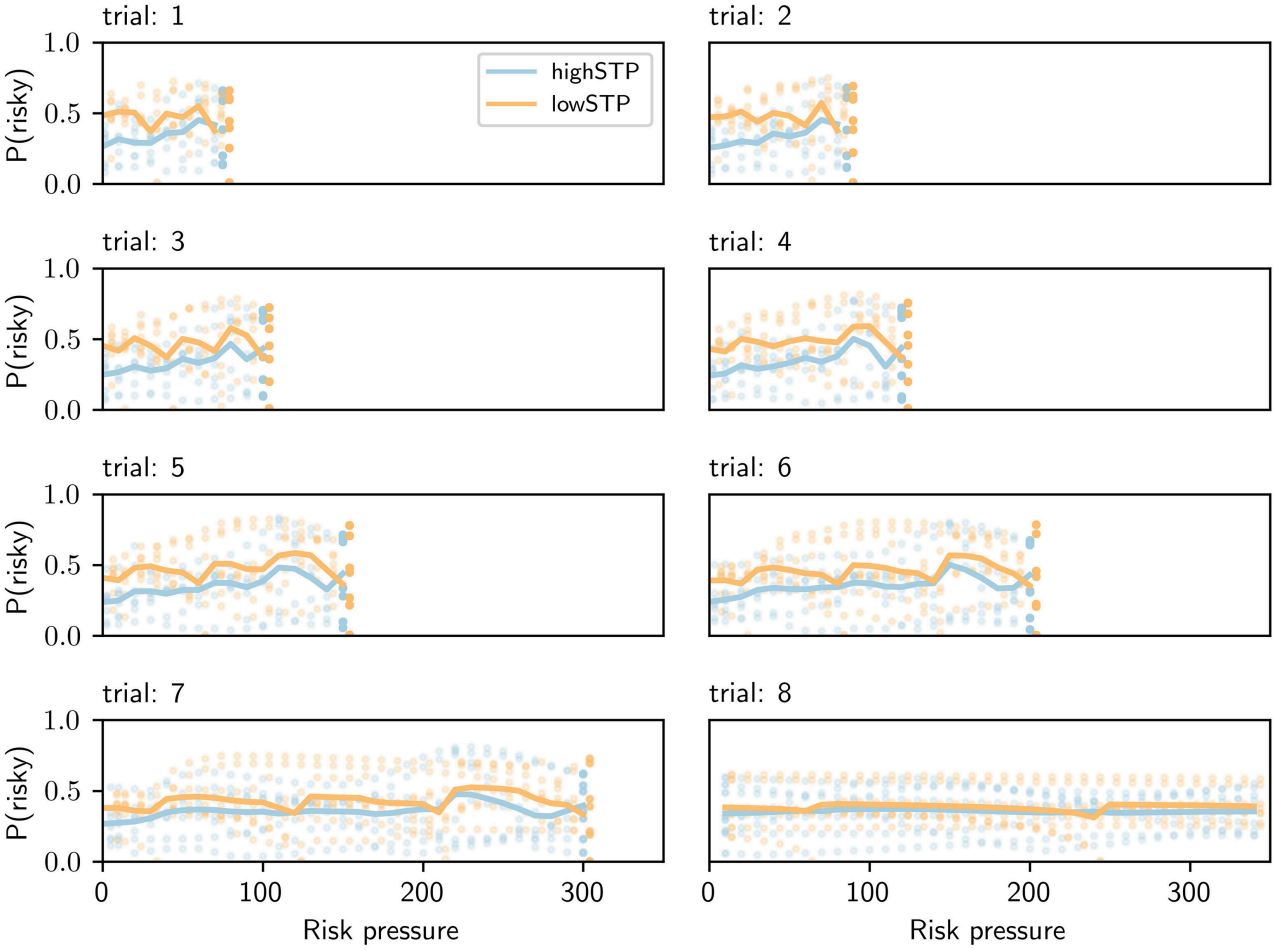
Risk aversion as a function of risk pressure and trial number. Each plot is for a different trial number, shown above each plot. Agent A, a representative of the high-STP group, is shown in blue. Agent B, a representative of the low-STP group, is shown in orange. We exposed both agents to all possible contexts in the task. Each single dot indicates the probability of choosing the risky choice for a single context. Orange dots were offset by 0.2 units to the right for visual clarity; averages (solid lines) were not offset. The solid line represents averages that were obtained for each value of risk pressure across all eight offers. For every trial, there is a maximum possible risk pressure (threshold divided by remaining trials); blank spaces on each plot are values of risk pressure beyond that maximum.

As shown above, the model-based approach allows us to have a trial-specific estimation of subjects' risk aversion. This enables us to separate trials not just by risk pressure as in Figure 7, but also by trial number, essentially creating a two-dimensional projection of the four-dimensional context (trial number, offered action pair, number of points that have been earned so far, and threshold). Such a separation, which can be seen in Figure 8 for agents A and B, revealed stereotyped trends in the adaptation of risk aversion to risk pressure as mini-blocks advanced.

Importantly, for each trial and risk pressure value, the vertical spread of the points is due to the available action pairs (eight of them), and not due to measurement errors, as there is no stochasticity in these estimated preferences.

A key feature of this presentation is the stereotyped curves, across trials, for average risk aversion adaptation that differ from trial to trial only in their length of spread across risk pressure values. This difference in length is caused by the fact that it is impossible to observe high risk pressures at the beginning of a mini-block, while on later trials the risk pressure may become quite large if the subject is missing a lot of points to reach the target. For any trial number, the high-STP subject type has a higher risk aversion than the low-STP subject type for the lowest values of risk pressure. For the largest values possible in the trial, however, this relationship is inverted, the low-STP subject type having now higher risk aversion than the other. This trend holds for most trials and slowly disappears toward the end of each mini-block. As can be seen in Figure 7, this phenomenon is lost, for both model-based and standard binning, when averaging across all trials and using the contexts observed by subjects.

To avoid biasing the estimates of Figure 8 with the specific set of contexts that any subject observed, we created a set of contexts which contains exactly one context for every combination of risk pressure, trial number and offered action pair. To make use of this set of contexts, we take advantage of the fact that our agents allow us to extrapolate how the corresponding subject would react to any context, even if it was not observed during the experimental session. For simplicity, we fixed the threshold to 595 for all contexts as we did not find evidence for differences in subject parameterizations for different conditions (see above).

This set of observations is unbiased in the sense that all possible observations are encountered exactly once. The importance of this can be seen in Figure 8: if for any value of risk pressure the action pairs with the highest probability of choosing the risky option had not been encountered by the subject, the average for this value of risk pressure (solid line) would be much lower. Conversely, if any one action pair had been encountered multiple times, the average would be skewed toward the probability of choosing the risky option for that action pair. The model-based approach is not susceptible to such biases. Critically, this bias is outside experimental control, as it depends on the subjects' choices during the experiment, which determine what risk pressure values are experienced for each action pair and trial number. Clearly, such a bias may impact the results and interpretation when using the binning method; see Discussion.

**Figure 9:**
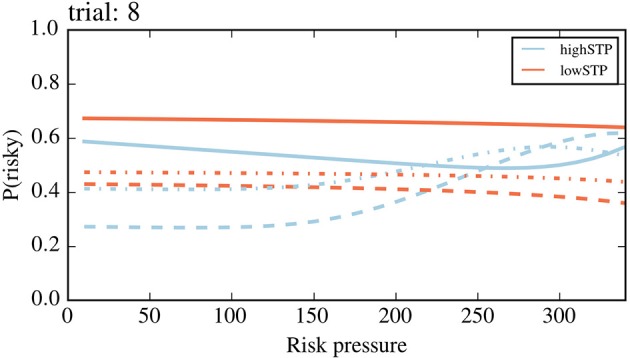
Probability of choosing the risky option on the last trial. Probability of choosing the risky option for the first four offers (action pairs) in Table 1, during the last trial, for both subject types (orange for high-STP, blue for low-STP). The four offers are plotted, each with a different type of line, (in the order of Table 1): solid, dashed, dash-dotted and dotted. Lines of the same type with two different colors represent the same offer for the two different subject types. The last four offers of Table 1 were removed for visual clarity. For example, the dashed lines represent the offer (safe: 180, risky: 260), where the blue dashed line shows that the high STP subject type chooses more often the risky choice once the risk pressure is above 180, i.e., there are more than 180 points missing. In contrast, the low STP subject type does not change much its choice behavior when offered this specific offer on the last trial.

Figure 8 also reveals a trend not visible in more coarse-grained descriptions, such as Figure 7: as mini-blocks progress, the average risk aversion across all values of risk pressure becomes flatter and more similar between the two subject types. This culminates in the last trial of the mini-block, where both average lines are very similar to each other and close to a flat line around medium values of risk pressure. We believe this to be a consequence of the smaller depth of future planning that a subject has to go through at each trial. In the last trial of the mini-block, when no future planning is necessary, the probability of choosing the risky choice peaks in very different places of risk pressure, for different action pairs, creating the flat average seen in the last trial (since this is the average across all action pairs). To make this clearer, in Figure 9, we plotted the probability of choosing the risky choice for four different offer pairs in the last trial. This plot contains the same data as the last panel (trial 8) of Figure 8, but different types of lines (e.g., dashed) are used instead of individual dots to aid in differentiating between action pairs, and the other four offers were removed to avoid visual clutter.

Figure 9 shows that, even when the difference in average risk aversion (averaged across all action pairs, for every risk pressure value) between the two agents is not large (as seen in Figure 8 for the 8th trial), the probability of choosing the risky option of a specific action pair can differ greatly between the two subject types. In general, for the high-STP subject type the probability of choosing the risky option for an action pair has steeper curves than for the low-STP subject type. This is partially due to the fact that the high-STP subject type is much more inclined to take the risky choice for any one given offer at risk pressure values in which the safe choice is not good enough to succeed. This is especially true if the probability of success for the safe offer is not much higher than the risky choice, e.g., for the 2nd offer (dashed line in Figure 9, safe offer: 180 points with probability 0.6, risky offer: 260 points with probability of 0.35; see Table 1).

Looking at the averages shown in Figure 8, it may appear as if the average differences in behavior (solid lines) between the two subject types can be explained by a potential difference in choice bias. This would shift the entire solid line up or down, if the choice bias were bigger or smaller than 1, respectively. However, it is evident from Figure 9 that this is not the case when looking in more detail at single offers: given the different shapes between the blue and orange curves, shifting any curve up or down (by adding a choice bias different from 1) would not make them similar across subject types.

Although we focused here on only two subject types to show principled subject-specific differences, note that these two subject types are representatives for the high-STP and the low-STP group. As we have shown in Figures 8, 9, the model-based approach enables a precise mapping between subject-specific goals and corresponding action preferences at the single-trial level for a concrete context experienced in a single trial. Here, we have used this mapping to infer from sequences of choices the internal goal function at the single-subject level.

## Discussion

To reveal adaptation of risk-taking behavior to changing context, we developed a model-based approach for inferring the risk aversion with subject- and trial-specific resolution. This allowed us to use the set of binary choices made by subjects to infer the preference that each subject had when making those decisions. The method uses the computational framework of active inference and is based on a decision-making model with free parameters that can be fitted to an individual subject's decisions. To show how this works in practice, we used a sequential decision-making task, in which subjects aim to achieve a goal over multiple trials. This task was designed to elicit changes in risk-taking behavior as the context changes in which the subject must make a decision.

We demonstrated how this model-based approach can be used to analyze behavior at a subject- and trial-specific resolution, which is generally not possible with standard binning methods due to limitations in the amount of data that is typically collected. Using clustering, we found that subjects can be divided into two groups, according to a key model parameter that describes the relative value that subjects give to increasing gains. We found that these two groups have different risk-taking behavior adaptation to changing context.

### Risk aversion adaptation

Traditionally, behavioral studies on risk aversion have focused on its fixed, context-independent component. Economic theories of decision making under risk, e.g., prospect theory (Kahneman and Tversky, 1979), as well as psychological experiments on probability discounting (Green et al., 1999), have focused on characterizing human choices in fixed, often hypothetical, contexts, and risk aversion is assumed not to change throughout the experiment.

Conversely, the dynamic, context-adapting aspect of risk aversion has seen much study in ecology, where it is established that risk aversion is not only a function of the given options, but also of the current needs and their urgency (McNamara and Houston, 1992). From ecology, risk-sensitive foraging theory (McNamara and Houston, 1992) emerged as a normative account of a foraging animal in an ever-evolving environment, which takes into account the changing needs of the foraging animal. The jump to the study of risk-taking behavior to human subjects has been made in the field of human behavioral ecology, where the tenets of theories, such as risk-sensitive foraging theory have been applied to human evolution; for an extensive review, see Winterhalder and Smith (2000).

Despite this, descriptive accounts of risk aversion as an adapting variable of human behavior have been more limited. Examples of studies that have focused on the dynamic, context-adapting side of risk aversion in human subjects are (Kolling et al., 2014), and (Schweighofer et al., 2006), the latter in the similar field of delay discounting.

The approach in Kolling et al. (2014) was a direct, data-driven measurement of risk aversion, which relied on binning enough decisions together to obtain reliable statistics, from which a preference for risk can be calculated. Such binning of distinct decisions is necessary in tasks with a large set of possible contexts since it becomes impossible to sample once, let alone enough times for a reliable estimation of a mean, any sizeable portion of the whole space. For example, in the task used by Kolling et al. (2014), the number of different contexts is in the order of tens of thousands, while the number of trials per subject was only 384.

In this work, we presented a model-based approach which is not affected by the size of the set of contexts and with which there is no need for a proxy variable nor binning decisions, since estimates on risk aversion can be obtained by fitting a decision-making model to the entire data set of each subject. This approach assumes that the experimentally-measured choices are sufficient to infer about an underlying choice mechanism with which one can extrapolate the remaining contexts not seen by the subject in the experiment. As the underlying choice mechanism we used the so-called active inference framework (Friston et al., 2015). The overall procedure resulted in an increased resolution, at the single-trial level, for the study of the adaptation of risk aversion to changing context. As a beneficial side effect, the model-based approach also avoids known statistical artifacts of binning approaches, e.g., hiding existing effects in the data (Siegler, 1987; Ashby et al., 1994; Estes and Maddox, 2005; Cohen et al., 2008).

### Subject classification and differences in behavior

Using the fitted model parameters, we found that subjects can be classified into two groups (see “Model parameters and fitting”). We found differences both when looking at subjects' preferences averaged across all trials, for which the differences are shown to be only evident in the low risk-pressure ranges (see Figure 7), and when looking at the more detailed per-trial analysis (see Figures 8, 9).

While it is difficult to discern differences in Figure 7 due to the averaging over trials (see “Adaptability of risk aversion”), Figures 8, 9 reveal the clearest differences. We found that there are clear differences between the choice behavior between the high-STP and low-STP subject type; for the high-STP subject type, risk aversion decreases as risk pressure increases, while for the low-STP subject type, the highest values of risk pressure see an increase in preference for risk.

The behavior of a high-STP subject type is to try and go above threshold. When more points are needed to reach the threshold (high risk pressure), more risk is taken. When few points are required (low risk pressure), risk is unnecessary and therefore avoided. We found that a low-STP group subject type shows a much weaker awareness of the threshold. It is an open question what exactly low-STP subjects try to achieve. A simple explanation may be that they were less motivated, or less goal-directed than the high-STP subject types to try and succeed in this specific task. Note that the model-based approach inferred these differences without an obvious reliance on subjects' performance: we did not find a significant performance difference between the two groups. We inferred the differences in subjects' internal representation of the task goal just by fitting the pattern of choices to the underlying active inference model. The finding that there was not a difference in performance between the two groups is possibly a consequence of the stochastic nature of the task; in future work, it may be useful to focus on less stochastic tasks to confirm the difference between the low- and high-STP groups also in terms of performance or other measures. We believe it is a strength of the proposed method that we can model the behavior of these subjects who do not necessarily follow the instructions to the letter but nevertheless perform well.

The lack of threshold awareness is noticeable when comparing the curves on the last (eighth) trial in Figures 8, 9. For the high-STP subject type, the probability of choosing the risky option for each offered action pair peaks at different values of risk pressure, depending on the action pair, which reveals threshold-awareness. The low-STP subject type instead shows little adaptation, at this last trial, of risk aversion to risk pressure.

The last trial also offers some insight into a feature of the model-based approach, as applied to this experimental task. Intuition suggests that the maximum probability of choosing the risky option should be very high in the last trial when the reward offered by the safe option is insufficient. However, the underlying heuristic of “the more the better” is present even for the high-STP subject type: this can be seen in Figure 6C where the goal shape of the high STP-group (blue solid line) has non-zero values even for a number of points below threshold. These non-zero values increase the usefulness of the safe choice even in cases where it is not enough to go above threshold, decreasing the preference for the better risky choice. This, coupled with the low probability of success with the risky choices, effects the lack of extreme preferences for the risky choice in the last trial seen in Figures 8, 9. Whether or not subjects' choices show the same behavior is rather difficult to determine because such cases occur rarely (around 1% of trials in our data set). Additionally, given the low number of occurrences, even a single safe choice made by a subject in these situations (e.g., by not paying attention or by miscalculation) throws off any estimate that is made based on these choices alone, or by the whole set of choices (as with our approach). Moreover, subjects had only a rather coarse-grained indicator of the number of missing points by a plotted bar as shown in Figure 1, so there may be cases where subjects were not entirely clear about whether a safe choice would be sufficient to reach the goal, or not. In fact, in the 1% of trials where a subject may obtain the target only by choosing the risky option, subjects chose the risky option only 75% of the times, which could explain the results seen in Figures 8, 9. It could prove interesting to explicitly model this observation noise in future studies in order to determine whether the inferred goal shapes better conform to the task's rules.

Starting from these initial results, the model-based approach can be used to generate predictions. For example, one could ask (i) how specific subjects will respond in specific but yet unseen contexts and whether this can be generalized and translated, using inferred parameters, to different experiments, and (ii) what specific contexts are those, for a specific experiment, that will show the most obvious differences between two groups of subject or, more generally, along a specific trait dimension. One can also use Bayesian model comparison to test alternative or extended versions of the current model. For example, the low percentage of risky choices observed in extreme contexts on the 8th trial might be caused by observation noise: subjects do not know exactly how many points they need to go above threshold and might therefore miscalculate the usefulness of the safe option. As a first approximation, the current model does not account for such observational noise; rather it calculates with absolute precision whether a given reward would suffice. Because of this, one way for the model to account for these decisions (safe choices in extreme cases) was to “soften” the shape of the goals, assuming that subjects found values below threshold acceptable, if not optimal, which gives a higher preference (likelihood) to the exponential family of goals over the other two.

### Describing a context with a proxy variable

Reducing a complex context into a one-dimensional proxy variable (such as Δ*V* or risk pressure) can bring with it loss of information. In the specific case of the task used here, as in many other tasks used in psychology, the context, i.e., the set of variables necessary to make a decision, is multi-dimensional. In many tasks, e.g., the urn task (FitzGerald et al., 2015), the set of all contexts is small enough that a proxy variable might not be necessary, or a natural proxy variable, such as trial number itself, can be used, as e.g., in Schweighofer et al. (2006). However, in other tasks as the one used here, in which the number of contexts is usually in the thousands, it is difficult to obtain veridical estimates on context-dependent risk aversion without using proxy variables that bin together dissimilar decisions.

With our model-based approach we do not need to use such a proxy variable for context; instead, the context is evaluated by the model by using all the information available to make a decision. This approach additionally revealed the effects of using a proxy variable. This can be seen in Figure 7 where, for each value of risk pressure, data points at different heights can be seen. These represent trials with different trial numbers, offered action pair, number of points and/or threshold, which nonetheless might have the same risk pressure. In standard binning approaches, these would be binned together to obtain summary statistics.

As a first attempt to validate the results from our model-based approach using standard binning methods, we compared the overall preference for the risky offer, averaged across all subjects and trials. A finer comparison was made in Figure 7, where we showed that binning the results using model-based binning yields curves of summary statistics that are very similar to those obtained with standard methods.

It is this similarity between the two methods that hints at important information being lost when binning many decisions: with the results from the binning method we could not conclude that any adaptation of risk aversion to risk pressure happens as we could not reject the null hypothesis of no risk adaptation for 25 out of 35 subjects. In contrast, our model-based approach reveals differences between stereotyped risk aversion adaptation curves that are clearly visible when looked at in a trial-specific fashion (Figure 8). Additionally, our method provided a single, subject-specific parameter value (i.e., STP, sensitivity to points) to explain the rather complex adaptation of risk aversion.

### Risk aversion

In this work we showed that differences in risk-taking behavior can be partially explained by differences in the way that subjects set goals for themselves. These internal goals are related to the goals of the task, as they were explained to the subjects, but are not necessarily the same as determined by the instructions.

The internal representation of goals sets not only the desired end states of a task, but also the valuations of these end states relative to each other (see Figure 6C). It is these relative values that can explain the inter-subject differences observed in the adaptation of risk aversion. For example, a subject with an internal goal of maximizing the number of points achieved at all costs could be less risk-prone that a subject who only wants to go above-threshold, due to the expected value of the safe choice is larger than that of the risky choice (see Figure 7). The effects of this internal representation of goals on risk aversion is highly contextual: in a given context, if two subjects have different internal goals, one subject might show a stronger preference for the risky offer than the other, while for another context, the opposite would be true. This is what differentiates this adaptive account of risk aversion from a trait-like account. Examples of this can be seen in Figure 9B, where differently-colored lines of the same action pair (e.g., dotted lines) cross each other.

To complement this adaptive account, we introduced the additional parameter of choice bias, which accounts for an overall preference for safe choices (risk aversion) or risky choices (risk proneness) by using prior probabilities for actions. In this work, we implemented a subject-specific, overall prior preference for (or against) the risky choice for any action pair. This choice bias is not informed about context or about the relative values and probabilities of success of the given choices. We found this simple, fixed bias to be an important parameter as evidenced by very strong evidence for it when comparing models. In this sense, the model presented in this work incorporates an adaptive risk aversion adaptation, which tailors responses to changing contexts, and a trait-like, non-adaptive risk aversion, which biases all responses toward or away from risk taking behavior. Similarly, in future work, additional biases, e.g., choice-supportive bias or the effect of previous successes with an offer, can be added to the generative model in a straightforward way, using model selection to identify those bias parameters that provide for better models of adaptive risk aversion.

## Data availability

All the experimental data, as well as the code necessary to reproduce our findings used in this manuscript is available online at doi: 10.25532/OPARA-6.

## Author contributions

Project conception was by DC and SK. Experimental design by DC, SK, and AS. Data gathering and programming of experiment by FO. Data analysis and modeling by DC, SK, and DM. Manuscript preparation by DC, SK, AS, FO, and DM.

### Conflict of interest statement

The authors declare that the research was conducted in the absence of any commercial or financial relationships that could be construed as a potential conflict of interest.

## References

[B1] Ashby F. G. Maddox W. T. Lee W. W. (1994). On the dangers of averaging across subjects when using multidimensional scaling or the similarity-choice model. Psychol. Sci. 5, 144–151. 10.1111/j.1467-9280.1994.tb00651.x

[B2] Basile A. G. Toplak M. E. (2015). Four converging measures of temporal discounting and their relationships with intelligence, executive functions, thinking dispositions, and behavioral outcomes. Front. Psychol. 6:728. 10.3389/fpsyg.2015.00728 26097462 PMC4456858

[B3] Beal M. J. (2003). Variational Algorithms for Approximate Bayesian Inference. Ph.D. thesis, Gatsby Computational Neuroscience Unit, University College London. Available online at: https://cse.buffalo.edu/faculty/mbeal/papers/beal03.pdf

[B4] Bornstein A. M. Khaw M. W. Shohamy D. Daw N. D. (2017). Reminders of past choices bias decisions for reward in humans. Nat. Commun. 8:15958. 10.1038/ncomms15958 28653668 PMC5490260

[B5] Camerer C. F. Hogarth R. M. (1999). The effects of financial incentives in experiments: a review and capital-labor-production framework. J. Risk Uncertain. 19, 7–42. 10.1023/A:1007850605129

[B6] Caraco T. Blanckenhorn W. U. Gregory G. M. Newman J. A. Recer G. M. Zwicker S. M. (1990). Risk-sensitivity: ambient temperature affects foraging choice. Anim. Behav. 39, 338–345. 10.1016/S0003-3472(05)80879-6

[B7] Caraco T. Martindale S. Whittam T. S. (1980). An empirical demonstration of risk-sensitive foraging preferences. Anim. Behav. 28, 820–830. 10.1016/S0003-3472(80)80142-4

[B8] Cartar R. V. Dill L. M. (1990). Why are bumble bees risk-sensitive foragers? Behav. Ecol. Sociobiol. 26, 121–127. 10.1007/BF00171581

[B9] Charness G. Gneezy U. Imas A. (2013). Experimental methods: eliciting risk preferences. J. Econ. Behav. Organ. 87, 43–51. 10.1016/j.jebo.2012.12.023

[B10] Choi S. Fisman R. Gale D. Kariv S. (2007). Consistency and heterogeneity of individual behavior under uncertainty. Am. Econ. Rev. 97, 1921–1938. 10.1257/aer.97.5.1921

[B11] Cohen A. L. Sanborn A. N. Shiffrin R. M. (2008). Model evaluation using grouped or individual data. Psychon. Bull. Rev. 15, 692–712. 10.3758/PBR.15.4.692 18792497

[B12] Daunizeau J. Friston K. J. Kiebel S. J. (2009). Variational Bayesian identification and prediction of stochastic nonlinear dynamic causal models. Phys. Nonlinear Phenom. 238, 2089–2118. 10.1016/j.physd.2009.08.002 19862351 PMC2767160

[B13] Economides M. Kurth-Nelson Z. Lübbert A. Guitart-Masip M. Dolan R. J. (2015). Model-based reasoning in humans becomes automatic with training. PLoS Comput. Biol. 11:e1004463. 10.1371/journal.pcbi.1004463 26379239 PMC4588166

[B14] Estes W. K. Maddox W. T. (2005). Risks of drawing inferences about cognitive processes from model fits to individual versus average performance. Psychon. Bull. Rev. 12, 403–408. 10.3758/BF03193784 16235625

[B15] Fantino E. Gaitan S. Kennelly A. Stolarz-Fantino S. (2007). How reinforcer type affects choice in economic games. Behav. Process. 75, 107–114. 10.1016/j.beproc.2007.02.001 17353099

[B16] FitzGerald T. H. Schwartenbeck P. Moutoussis M. Dolan R. J. Friston K. (2015). Active inference, evidence accumulation and the urn task. Neural Comput. 27, 306–328. 10.1162/NECO_a_00699 25514108 PMC4426890

[B17] Freedman D. Diaconis P. (1981). On the histogram as a density estimator: L2 theory. Z. Für Wahrscheinlichkeitstheor. Verw. Geb. 57, 453–476. 10.1007/BF01025868

[B18] Friston K. Rigoli F. Ognibene D. Mathys C. Fitzgerald T. Pezzulo G. (2015). Active inference and epistemic value. Cogn. Neurosci. 6, 187–214. 10.1080/17588928.2015.1020053 25689102

[B19] Goutte C. Toft P. Rostrup E. Nielsen F. Å. Hansen L. K. (1999). On clustering fMRI time series. Neuroimage 9, 298–310. 10.1006/nimg.1998.0391 10075900

[B20] Green L. Myerson J. Ostaszewski P. (1999). Amount of reward has opposite effects on the discounting of delayed and probabilistic outcomes. J. Exp. Psychol. Learn. Mem. Cogn. 25, 418–427. 10.1037/0278-7393.25.2.418 10093208

[B21] Hertwig R. Ortmann A. (2001). Experimental practices in economics: a methodological challenge for psychologists? Behav. Brain Sci. 24, 383–403; discussion 403–451. Available online at: http://psycnet.apa.org/record/2001-18911-001 11682798 10.1037/e683322011-032

[B22] Hey J. D. Orme C. (1994). Investigating generalizations of expected utility theory using experimental data. Econometrica 62, 1291–1326. 10.2307/2951750

[B23] Houston A. I. Fawcett T. W. Mallpress D. E. W. McNamara J. M. (2014). Clarifying the relationship between prospect theory and risk-sensitive foraging theory. Evol. Hum. Behav. 35, 502–507. 10.1016/j.evolhumbehav.2014.06.010

[B24] Jones E. Oliphant T. Peterson P. (2001). SciPy: Open Source Scientific Tools for Python. Available online at: http://www.scipy.org/

[B25] Kacelnik A. Bateson M. (1996). Risky theories—the effects of variance on foraging decisions. Integr. Comp. Biol. 36, 402–434.

[B26] Kahneman D. Tversky A. (1979). Prospect theory: an analysis of decision under risk. Econometrica 47, 263–291. 10.2307/1914185

[B27] Kass R. E. Raftery A. E. (1995). Bayes factors. J. Am. Stat. Assoc. 90, 773–795. 10.1080/01621459.1995.10476572

[B28] Kellen D. Pachur T. Hertwig R. (2016). How (in)variant are subjective representations of described and experienced risk and rewards? Cognition 157, 126–138. 10.1016/j.cognition.2016.08.020 27620961

[B29] Kolling N. Behrens T. E. Mars R. B. Rushworth M. F. (2012). Neural mechanisms of foraging. Science 336, 95–98. 10.1126/science.1216930 22491854 PMC3440844

[B30] Kolling N. Wittmann M. Rushworth M. F. S. (2014). Multiple neural mechanisms of decision making and their competition under changing risk pressure. Neuron 81, 1190–1202. 10.1016/j.neuron.2014.01.033 24607236 PMC3988955

[B31] Lloyd S. (1982). Least squares quantization in PCM. IEEE Trans. Inf. Theory 28, 129–137. 10.1109/TIT.1982.1056489

[B32] Locey M. L. Jones B. A. Rachlin H. (2011). Real and hypothetical rewards. Judgm. Decis. Mak. 6, 552–564. 22582110 PMC3348706

[B33] Mather M. Shafir E. Johnson M. K. (2003). Remembering chosen and assigned options. Mem. Cognit. 31, 422–433. 10.3758/BF03194400 12795484

[B34] McNamara J. M. Houston A. I. (1992). Risk-sensitive foraging: a review of the theory. Bull. Math. Biol. 54, 355–378. 10.1007/BF02464838

[B35] Mobbs D. Hassabis D. Yu R. Chu C. Rushworth M. Boorman E.. (2013). Foraging under competition: the neural basis of input-matching in humans. J. Neurosci. 33, 9866–9872. 10.1523/JNEUROSCI.2238-12.2013 23739983 PMC3865496

[B36] Moran R. Pinotsis D. A. Friston K. (2013). Neural masses and fields in dynamic causal modeling. Front. Comput. Neurosci. 7:57. 10.3389/fncom.2013.00057 23755005 PMC3664834

[B37] Myerson J. Green L. Scott Hanson J. Holt D. D. Estle S. J. (2003). Discounting delayed and probabilistic rewards: processes and traits. J. Econ. Psychol. 24, 619–635. 10.1016/S0167-4870(03)00005-9

[B38] Rieskamp J. (2008). The probabilistic nature of preferential choice. J. Exp. Psychol. Learn. Mem. Cogn. 34, 1446–1465. 10.1037/a0013646 18980407

[B39] Schwartenbeck P. FitzGerald T. H. Mathys C. Dolan R. Friston K. (2015). The dopaminergic midbrain encodes the expected certainty about desired outcomes. Cereb. Cortex 25, 3434–3445. 10.1093/cercor/bhu159 25056572 PMC4585497

[B40] Schwarz G. (1978). Estimating the dimension of a model. Ann. Stat. 6, 461–464. 10.1214/aos/1176344136

[B41] Schweighofer N. Shishida K. Han C. E. Okamoto Y. Tanaka S. C. Yamawaki S.. (2006). Humans can adopt optimal discounting strategy under real-time constraints. PLoS Comput. Biol. 2:e152. 10.1371/journal.pcbi.0020152 17096592 PMC1635539

[B42] Shead N. W. Hodgins D. C. (2009). Probability discounting of gains and losses: implications for risk attitudes and impulsivity. J. Exp. Anal. Behav. 92, 1–16. 10.1901/jeab.2009.92-1 20119519 PMC2707142

[B43] Siegler R. (1987). The perils of averaging data over strategies–an example from childrens addition. J. Exp. Psychol. Gen. 116, 250–264. 10.1037/0096-3445.116.3.250

[B44] Walasek L. Stewart N. (2015). How to make loss aversion disappear and reverse: tests of the decision by sampling origin of loss aversion. J. Exp. Psychol. Gen. 144, 7–11. 10.1037/xge0000039 25485606 PMC4312134

[B45] Winterhalder B. Smith E. A. (2000). Analyzing adaptive strategies: human behavioral ecology at twenty-five. Evol. Anthropol. Issues News Rev. 9, 51–72. 10.1002/(SICI)1520-6505(2000)9:2<51::AID-EVAN1>3.0.CO;2-7

[B46] Xu S. Pan Y. Qu Z. Fang Z. Yang Z. Yang F.. (2018). Differential effects of real versus hypothetical monetary reward magnitude on risk-taking behavior and brain activity. Sci. Rep. 8:3712. 10.1038/s41598-018-21820-0 29487303 PMC5829218

