# Supplementary material for: Context-Dependent Risk Aversion: A Model-Based Approach
Source: Front Psychol. 2018 Oct 26;9:2053. doi: 10.3389/fpsyg.2018.02053 (PMC6212575; doi:10.3389/fpsyg.2018.02053)
Supplement: Supplementary file 1 [file Table_1.PDF]

Subject	MDM	Bias	STP	Subject	MDM	Bias	STP
**0**	3.1	1.2	99.0	18	10.0	0.6	57.0
**1**	10.0	0.6	99.0	19	4.1	1.2	27.0
**2**	20.0	0.7	29.0	20	10.0	0.6	99.0
**3**	5.0	0.9	99.0	21	0.2	0.8	81.0
**4**	1.6	0.8	95.0	22	1.7	0.9	5.0
**5**	0.1	0.7	5.0	23	10.0	0.6	93.0
**6**	10.0	0.6	57.0	24	10.0	0.6	83.0
**7**	1.0	0.7	5.0	25	20.0	0.6	31.0
**8**	0.8	1.2	99.0	26	10.0	0.6	99.0
**9**	10.0	0.6	99.0	27	0.1	0.7	99.0
**10**	10.0	0.6	51.0	28	10.0	1.1	99.0
**11**	2.2	0.6	5.0	29	5.0	0.6	99.0
**12**	5.0	1.1	99.0	30	0.1	1.2	99.0
**13**	0.1	1.2	99.0	31	5.0	0.6	9.0
**14**	3.9	0.6	99.0	32	0.4	1.1	5.0
**15**	5.0	0.6	79.0	33	0.8	0.8	5.0
**16**	5.0	1.2	91.0	34	0.3	0.9	5.0
**17**	20.0	1.2	91.0				

Sup. table 1. **Inferred parameters for all subjects**. List of the model parameters and the values inferred for each subject, using their behavioral data; for all subjects, the exponential family of goals is plotted, where STP (sensitivity to points; see main text) translates into the coefficient of the exponent. This relates to the section ‘SUBJECT-SPECIFIC PARAMETER VALUES’ of the main text.
