# Supplementary material for: Context-Dependent Risk Aversion: A Model-Based Approach
Source: Front Psychol. 2018 Oct 26;9:2053. doi: 10.3389/fpsyg.2018.02053 (PMC6212575; doi:10.3389/fpsyg.2018.02053)
Supplement: Supplementary file 2 [file Image_1.PDF]

Supplementary Information

SUPPLEMENTARY INFORMATION

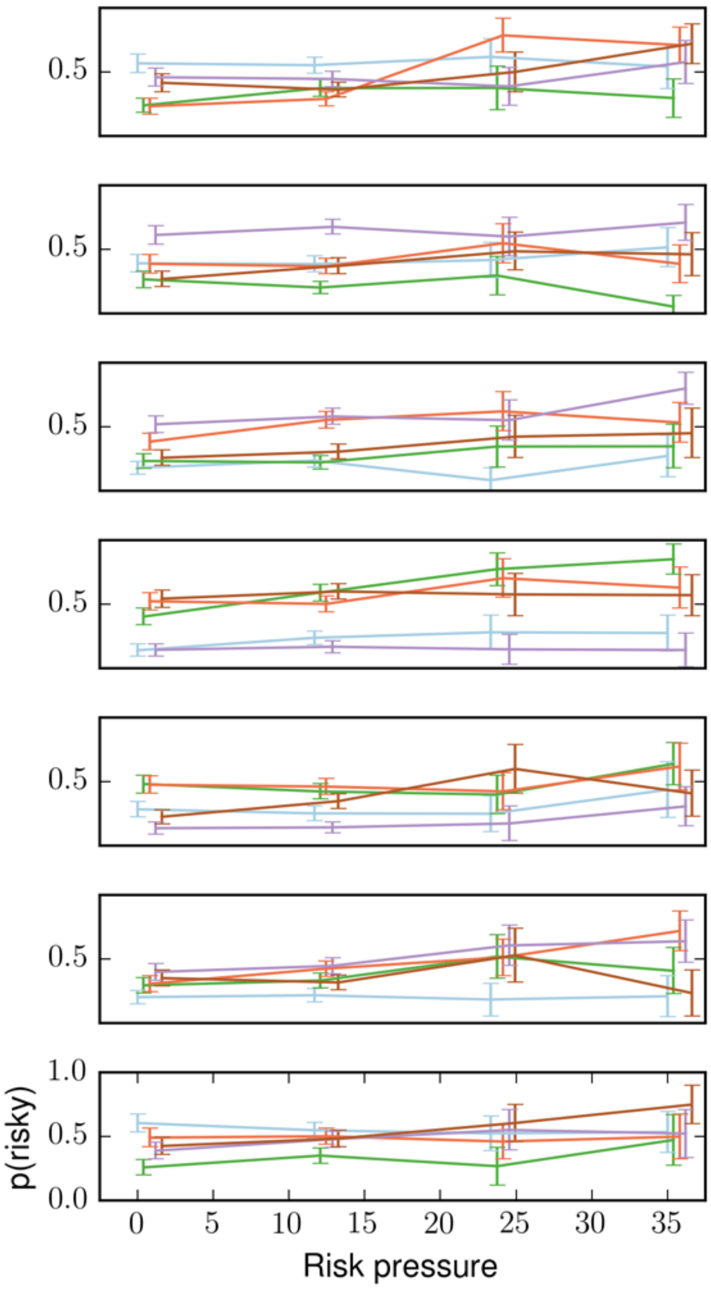

Sup. Fig. 1. **Risk-aversion as a function of risk pressure**. As in Figure 2B of the main text, the probability of choosing the risky option is calculated by binning all decisions around four values of risk pressure and calculating the proportion of risky choices to all choices. Each line represents one subject, separated into seven plots for clarity. All subjects were binned using the same bin positions; however, the lines were offset slightly in terms of risk pressure to avoid overlap in the confidence intervals.
