# Supplementary material for: Context-Dependent Risk Aversion: A Model-Based Approach
Source: Front Psychol. 2018 Oct 26;9:2053. doi: 10.3389/fpsyg.2018.02053 (PMC6212575; doi:10.3389/fpsyg.2018.02053)
Supplement: Supplementary file 3 [file Image_2.PDF]

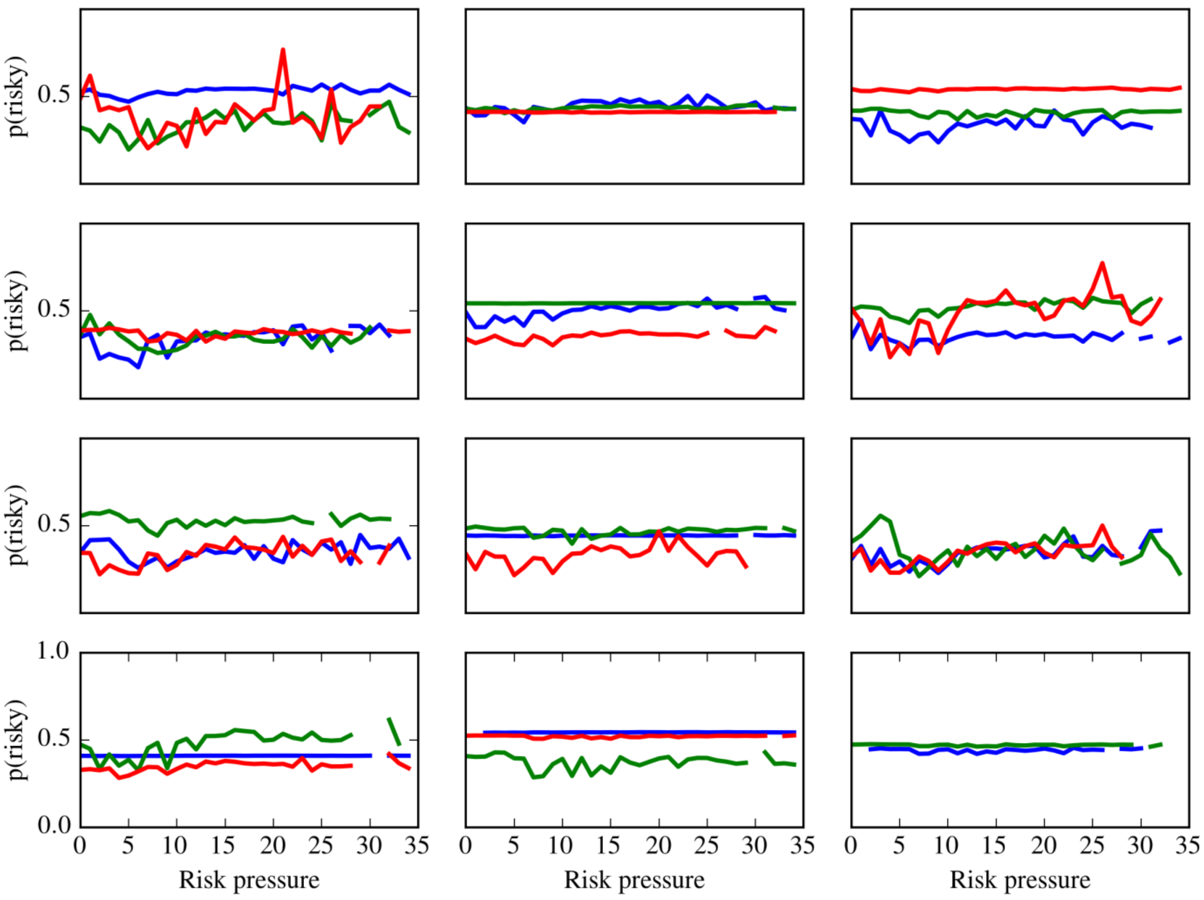

Sup. fig. 2. **Estimates of risk aversion from our model**. As in Figure 6, risk aversion as estimated by our model-based approach. All 35 subjects are shown (one line per subject). They were split into 12 plots for clarity, with each plot including three subjects (except for the bottom-right plot, which only has two).
